# Gas–Responsive Metal–Organic Frameworks for Adaptive Thermal Energy Storage with Tunable Charge–Discharge Temperatures

**DOI:** 10.1021/jacs.6c07093

**Published:** 2026-07-06

**Authors:** María Gelpi, David González-Novo, Lorena Alonso-Marañón, Javier García-Ben, Álvaro Baaliña, Sonia Zaragoza, Julian Walker, Charles James McMonagle, Socorro Castro-García, María Antonia Señarís-Rodríguez, Manuel Sánchez-Andújar, Juan Manuel Bermúdez-García

**Affiliations:** † CICA—Interdisciplinar Center of Chemistry and Biology, Department of Chemistry, Faculty of Sciences, 16737 Universidade da Coruna, Campus de Elviña 15071 A Coruna, Spain; ‡ Energy Engineering Research Group, University Institute of Maritime Studies, Nautical Sciences and Marine Engineering Department, Escuela Técnica Superior de Náutica y Máquinas (ETSNM), Universidade da Coruna, Paseo de Ronda 51, 15011 A Coruna, Spain; § Universidade da Coruna (UDC), Ferrol Industrial Campus, CITENI, 15403 Ferrol, A Coruna, Spain; ∥ Department of Materials Science and Engineering, 205199 Norwegian University of Science and Technology, 7491 Trondheim, Norway; ⊥ Swiss−Norwegian Beamlines, European Synchrotron Radiation Facility 38043 Grenoble, France

## Abstract

One of the major challenges for thermal energy storage (TES) systems based on phase change materials (PCMs)—a key technology for energy decarbonization—is achieving dynamical tunability of their charge and discharge temperatures (*T*_charge_, *T*_discharge_). This capability would enable a single material to operate effectively across varying ambient conditions (e.g., different times of day, seasons, or climates) without requiring the use of multiple TES materials. In this work, we present a gas-responsive TES solution based on breathing caloric MOFs, particularly MOF-508b, in which transition temperatures can be widely tuned from −30 to 120 °C (243 to 393 K) by adjusting the surrounding CO_2_ pressure from 5 to 25 bar under isobaric conditions. More importantly, we demonstrate that this method allows precise control over the thermal hysteresis (|*T*_charge_ – *T*_discharge_|) and can even reverse the transition temperatures (*T*_charge_ < *T*_discharge_), which could be highly advantageous for temperature-driven TES applications. These findings pave the way for adaptive thermal technologies in which a single material can respond to different ambient temperatures and diverse storage needs, from cold storage to medium–high-temperature applications. We anticipate that this gas-responsive behavior is not exclusive to MOF-508b and encourage the exploration of other materials exhibiting similar tunable characteristics for adaptive TES applications.

## Graphical abstract

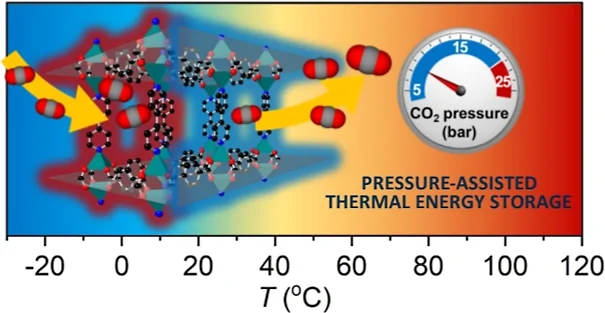

## 1 Introduction

Thermal energy storage (TES) is considered a cornerstone in the current transition toward a climate-neutral, more energy-efficient, and decarbonized economy.^1^ Such technologies can not only enable the storage of surplus thermal energy from different domestic and/or industrial sources but also allow the decoupling of energy supply and demand, facilitating a more efficient use of renewable energies. In fact, recent reports highlight that TES can reduce around 1800 TWh of energy generated from fossil fuels and 513 Mt of carbon emissions per year.^2^

These systems are gaining special relevance for decreasing the energy needed for heating and cooling. As a matter of example, this sector already represents 50% of final energy consumption in the EU, even ahead of transport.^3^ In that regard, the current energy transition scene is accelerating the development of new self-sufficient systems for smart buildings that can store energy surplus for later use.^4^

In addition, TES technologies can provide support for maintaining uninterrupted cold chains for temperature-sensitive goods (such as food or medicine supplies) and for thermal management of electronic devices and data centers.^5^

Along the years, different TES technologies have been developed and/or integrated in the market based on different types of storage media, including (i) substances with large sensible heat, (ii) phase change materials (PCMs) with large latent heat due to solid–liquid and solid–solid transitions, and (iii) sorption materials with large sorption heat due to chemisorption and/or physisorption processes.

Substances with large sensible heat—such as sand, water, or other liquids—only rely on their specific heat capacity (*C*_*p*_) to store thermal energy. Their technological integration is simple, but their storage capacity is limited.^6,7^

In that regard, PCMs can store significantly larger amounts of thermal energy due to large enthalpy changes exhibited during the phase transitions.^2,8^ Most of the PCMs in the market today rely on thermally driven solid–liquid phase transitions (paraffins, fatty acids, and salt hydrates),^9^ which are very energetic but pose technological challenges associated with overcooling, incongruent melting, mechanical fatigue induced by large volume changes, and/or liquid leaks and losses. Trying to overcome those drawbacks, many efforts have been devoted to develop PCMs with solid–solid phase transitions (hybrid perovskites, plastic crystals, and polymers).^9^ Nevertheless, their thermal changes must still be improved.

On the other hand, sorption TES materials are arising as an innovative solution with large storage capacity related to sorption heat processes in different sorbate–sorbent pairs (such as water–zeolites or water–NaOH).^10^ However, the technological integration of these materials exhibits larger complexity and generally requires operation under vacuum conditions (0.01–0.05 bar) to regenerate the sorbents,^10^ which hinders the heat transfer.

Furthermore, an important and common challenge in TES technologies is storing the energy for prolonged periods. In that context, Tokoro et al. proposed in 2015 a pressure-assisted TES mechanism that allows storing thermal energy for a long period in Ti_3_O_5_ under a temperature-driven solid–solid phase transition until the application of 600 bar triggers the energy release.^11^ Also, Zhang et al. observed that the barocaloric material NH_4_SCN can store thermal energy upon pressurization of 1000 bar, which can be maintained and later released upon depressurization.^12^

Another critical and major drawback to solve is the limited tunability of the operating temperatures of a given TES material, as highlighted by A. Henry et al. in their report on the “*Five thermal energy grand challenges for decarbonisation”*.^13^ Indeed, up to date, the reported PCMs for TES applications (pressure-assisted or not) must operate in a very narrow and rigid range close to the temperatures of their phase transitions, namely, their charge temperature, *T*_charge_ (transition temperature that gives rise to the heat storage), and discharge temperature, *T*_discharge_ (reverse transition temperature that gives rise to the heat release). As a result, the variations of temperatures over time (daily and seasonal) require the use of several TES materials, which increases technological costs.^13^

For that reason, there is an emerging interest in the study of dynamic tunability of PCM transition temperatures by using different stimuli (pressure, electric field, magnetic field, and even modulation of the ionic media) to adapt to temperature swings, either diurnal or even seasonal.^14^ These works propose to dynamically modulate the transition temperature of TES materials to match it with the ambient temperature at any moment.^14^ Nevertheless, these studies also highlight that the required magnitude of the applied external stimuli is still very large and hinders practical application.^14^

In the search for new materials that can exhibit enhanced TES capacities, metal–organic frameworks (MOFs) are attracting increasing interest among the scientific and industrial communities. In that regard, recent studies have identified MOFs with reversible solid–liquid transitions of interest for TES based on their large latent heat^15^ and also MOFs with large sorption heat when combined with water and/or alcohols of interest for sorption TES technologies.^16^ Even more recently, MOFs have been proposed to confine PCMs, where the transition temperature can be easily modulated by controlling the hydrogen bonding.^17^

In parallel, our group has recently reported that flexible metal–organic frameworks (MOFs), including MIL-53­(Al) (Al­(OH)­(bdc), bdc = 1,4-benzenedicarboxylate), and MOF-508b ([Zn_2_(bdc)_2_(bpy)], bdc = 1,4-benzenedicarboxylate, bpy = 4,4′- bipyridine), can exhibit very large thermal changes named breathing-caloric effects.^18,19^ These effects arise from CO_2_ adsorption/desorption, which triggers solid–solid transitions between a narrow-pore phase and a large-pore phase—phenomena known as breathing transitions.^20–22^

We have shown that the continuous pressure cycling of these MOFs under isothermal conditions near room temperature already produces exo- and endothermic processes of great interest for traditional heating and cooling applications, such as fridges, air conditioners, or heat pumps.^18,19^

Now, in the present work, we study for the first time the thermal energy storage capacity of these breathing-caloric MOFs (particularly of MOF-508b) for pressure-assisted thermal energy storage. For that purpose, and differently from previous investigations, we have performed structural, calorimetric, and sorption studies upon continuous temperature cycling under different isobaric conditions.

Our research has revealed three significant discoveries: (i) MOF-508b displays very energetic breathing-caloric effects triggered not only by pressure (as previously reported)^19^ but also by temperature; (ii) the charge (heat storage) and discharge (heat release) temperatures can be widely tuned from −30 to 120 °C (243–393 K) just by setting different isobaric CO_2_ conditions under low pressures (5–25 bar); and (iii) the thermal hysteresis (|*T*_charge_ – *T*_discharge_|) can also be modulated and even reversed under different isobaric conditions, so the *T*_discharge_ can be shifted well above the *T*_charge_.

These findings represent the first report of such behavior in any temperature-triggered thermal energy storage (TES) material. They introduce a novel approach for designing highly adaptable TES technologies capable of responding to varying diurnal or seasonal temperatures (e.g., winter to summer) and diverse storage needs (from cold storage to medium- high temperature applications). This adaptability is achieved simply by adjusting isobaric conditions within the same material in a straightforward and reproducible process.

## 2 Results and Discussion

### 2.1 Thermally Induced Breathing Transitions under Different Isobaric Conditions in MOF-508b

The MOF-508b was synthesized as a pure polycrystalline powder, as observed by powder X-ray diffraction (PXRD), ^1^H NMR, and ^13^C NMR (see Figures S1–S3 of Supporting Information and Experimental Section). Differently from previous variable-pressure studies under different isothermal conditions,^19,22,23^ in this work, we perform variable-temperature studies under different isobaric conditions. For that purpose, we have combined synchrotron powder X-ray diffraction (SPXRD), thermogravimetric analysis (TGA), and differential scanning calorimetry (DSC).

From a general point of view, under room conditions of pressure and temperature, the crystal structure of MOF-508b was reported to be formed by 2 interpenetrated subnetworks, where each subnetwork presents planes of Zn paddle-wheel nodes linked by bdc ligands and interconnected through bpy pillars (Figure 1a,c).^22–24^ In this crystal structure, the metallic nodes of one subnetwork partially or totally block the cavities of the other one, hindering any gas adsorption.

**Figure 1:**
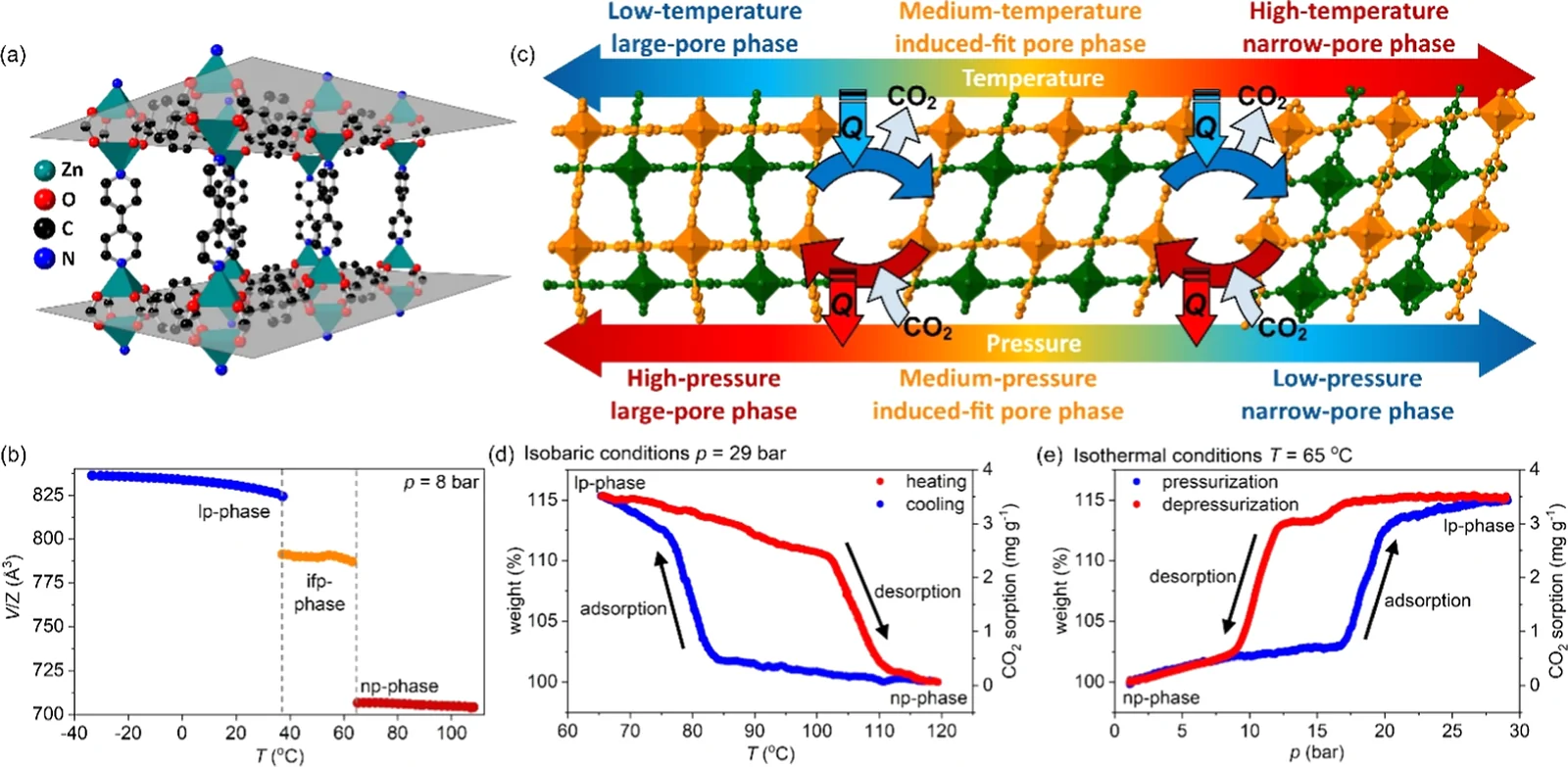
Structural and sorption data of MOF-508b: (a) Representation of one of the interpenetrated subnetworks, where gray planes represent bdc-coordinating ligands between the Zn-nodes and bpy pillars interconnect those planes. (b) Unit cell volume of the principal phases upon heating under the isobaric CO_2_ pressure of 8 bar, as obtained by SPXRD, where the transition temperatures marked with dashed lines correspond to the temperature above which the presence of the lp-phase and ifp-phase, respectively, is negligible. (c) Schematic representation (based on CIF files of ref 22.) of the lp-phase, ifp-phase, and np-phase that can be induced as a function of temperature (as observed in this work) and also by pressure (as previously reported).^22^ (d) VT-TGA curves from 65 to 120 °C (338–393 K) under CO_2_ isobaric conditions of 29 bar, showing CO_2_ desorption/adsorption upon heating/cooling due to phase transitions between the lp-phase and np-phase. (e) VP-TGA curves from 1 to 29 bar under isothermal conditions of 65 °C (338 K), showing CO_2_ adsorption/desorption upon pressurization/depressurization due to phase transitions between the np-phase and lp-phase.

Our studies with SPXRD upon temperature increase (from −30 to 120 °C) at different isobaric conditions reveal 2 consecutive solid–solid phase transitions (see Figure 1b and Figures S4–S6 of Supporting Information). When heating under CO_2_ isobaric conditions, these transitions occur from a majority large-pore polymorph (lp-phase) to a majority intermediate temperature-induced-fit pore polymorph (ifp-phase) and finally to a high-temperature narrow-pore phase (np-phase). The predominant phase for each region coexists with smaller amounts of the previous phases, probably due to kinetic effects related to the experimental conditions. The transition temperatures are selected as the minimum temperatures above which the presence of either the lp-phase or ifp-phase, respectively, turns to be minimal.

The three observed polymorphs can be related to those previously reported by L. Barbour et al.,^22^ even if from pressure-induced phase transitions rather than temperature-driven transitions. Accordingly, the here-reported low-temperature lp-phase will correspond to the high-pressure lp-phase described by Barbour and co-workers, while the here-presented high-temperature np-phase corresponds to the low-pressure np-phase described by those authors (see Figure 1c).

Considering that the previously reported pressure-induced transitions are related to CO_2_ sorption processes, we anticipated that similar processes could be responsible for the here-observed thermally induced transitions.

Traditionally, most sorption studies on MOFs have been focused on pressure swing adsorption (PSA) under variable pressure at different isothermal conditions. Nevertheless, for the evaluation of the thermally induced breathing transitions for thermal energy storage, we find it necessary to also address the temperature swing adsorption (TSA).

Accordingly, in this study, we performed both isobaric and isothermal sorption studies using a singular technique scarcely used in the characterization of sorption materials: variable-temperature (VT) and variable-pressure (VP) TGA under a CO_2_ atmosphere.

Our VT-TGA data under isobaric conditions (*p* = 29 bar) show that the sample mass decreases upon heating while increasing back to the initial value upon cooling (Figure 1d). This behavior can be explained by a CO_2_ release occurring during the transformation from the lp-phase to the np-phase, as also observed by SPXRD (Figure 1b). Meanwhile, CO_2_ is adsorbed back during the reverse transformation upon cooling. In the same line, our VP-TGA data under isothermal conditions (*T* = 65 °C) reveal that CO_2_ pressurization provokes a weight gain, which is subsequently decreased when depressurizing (Figure 1e). This is in full agreement with previously reported isothermal sorption studies.^22,23^ In both VT-TGA and VP-TGA curves, a small step can be observed at ∼95 °C and at ∼13 bar, respectively, which corresponds to the intermediate ifp-phase. Furthermore, in both VT- and VP-TGA studies, the maximum CO_2_ adsorption capacity of MOF-508b in the lp-phase is ∼3.5 mmol g^–1^, which perfectly aligns with capacity values reported in the literature.^22,23^

In turn, we can conclude that both thermally induced and pressure-induced phase transitions can lead to a large CO_2_ adsorption related to the phase transition between the np-phase and the lp-phase.

In addition, cycling studies over time reveal that the sorption processes are fully reversible upon heating and cooling cycles (see Figure S7 of Supporting Information). Furthermore, our VT-TGA studies also indicate that the thermal stability of MOF-508b increases under higher CO_2_ isobaric pressures, which would also benefit potential technological applications (see Figure S8 of Supporting Information). Another advantage for potential practical applications is that even if many MOFs are known to be unstable under water exposure, MOF-508b remains stable even under 90% RH conditions.^25,26^

We have also performed variable-temperature differential scanning calorimetry (VT-DSC) at different isobaric conditions, using different pressures from 5 to 25 bar (see Figure 2a) and starting at a low temperature (−30 °C). Upon temperature increase, two consecutive peaks appear that can be related to the phase transitions from the lp-phase to the ifp-phase and from the ifp-phase to the np-phase and are reversed when cooling back, in good agreement with the results of TGA and SPXRD.

**Figure 2:**
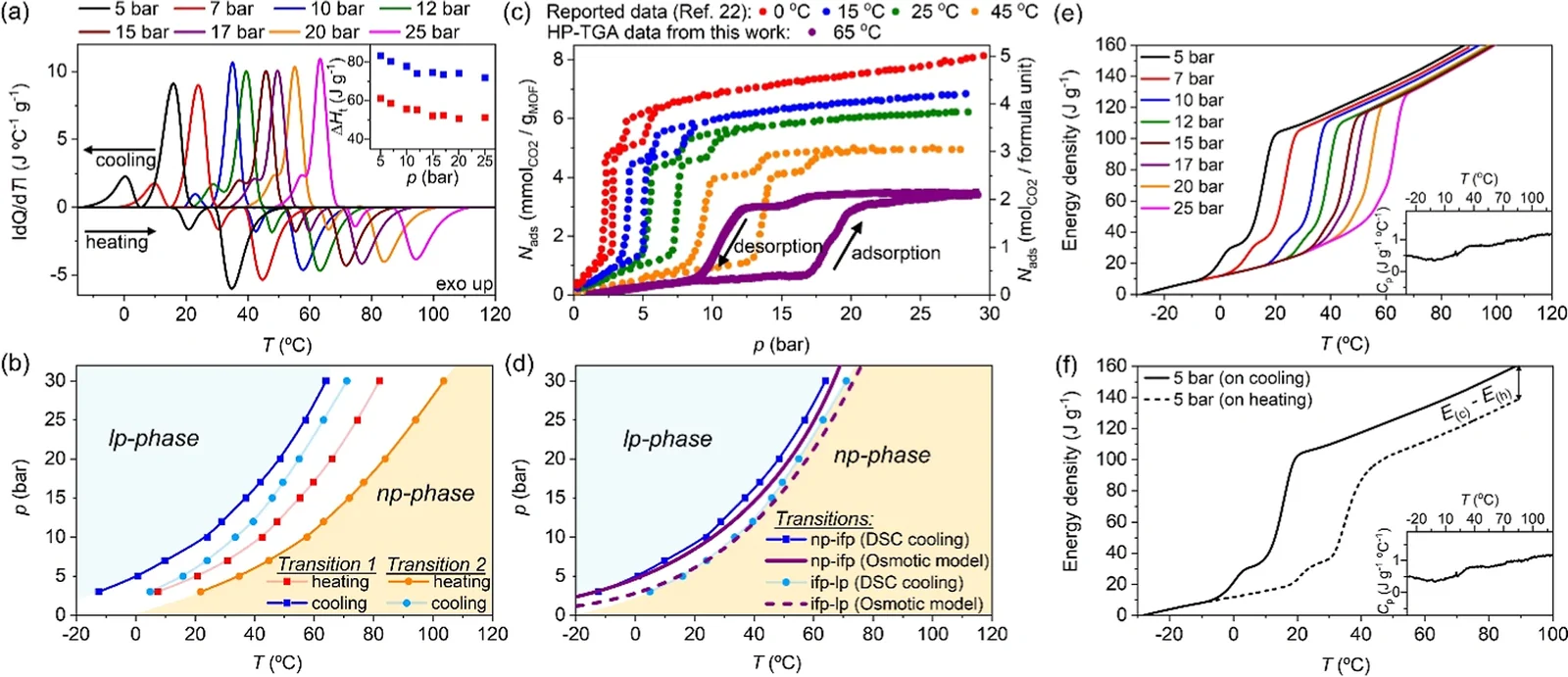
(a) VT-DSC curves under different isobaric pressures (5–25 bar). Inset: total enthalpy change Δ*H*_t_ of the two combined transitions on heating (red) and cooling (blue). (b) *p*–*T* phase diagram obtained from the peaks’ maximum of the VT-DSC curves, showing the large-pore (lp-phase), induced-fit pore (ifp-phase), and narrow-pore (np-phase) stability regions. (c) CO_2_ adsorption isotherms under variable pressure at different isothermal conditions for MOF-508b extracted from reference 22 (data from 0 to 45 °C) and complemented with our HP-TGA values (data for 65 °C). (d) Comparison of the *p*–*T* phase diagram of MOF-508b as obtained by the osmotic ensemble model from adsorption isotherms and by VT-DSC at different pressures upon cooling. (e) Energy density calculated from the contributions of the enthalpy change Δ*H*_t(c)_ and specific heat capacity *C*_p_ at different pressures. (f) Comparison of the observed difference for the energy density at 5 bar upon cooling and upon heating, which can be attributed to gas trapping interactions, desorption kinetics, and thermal hysteresis effects (see main text for details).

The here-reported findings also demonstrate that the surrounding CO_2_ pressure affects the release of CO_2_ guests from the nanopores of MOF-508b, causing the phase transition to occur at higher temperatures as the external CO_2_ pressure increases. As an interesting result, the heat charge/discharge temperatures (*T*_charge_/*T*_discharge_) can be tuned by approximately 60 °C when modulating the CO_2_ pressures, even in the very narrow range of 5–25 bar (Figure 2a). In addition, at higher isobaric conditions, the two consecutive peaks progressively merge, suppressing the ifp-phase.

From these VT-DSC experiments, we can define a pressure–temperature (*p*–*T*) phase diagram with three regions corresponding to the different polymorphs (Figure 2b).

Remarkably, the transition temperature of the MOF-508b shows a very large pressure dependence −d*T*_t_/d*p*– , a parameter usually known as the barocaloric coefficient in the case of barocaloric materials. It is worth noting that, in such barocaloric materials, the modulation is generally linear with values of d*T*_t_/d*p* ≤ 40 K kbar^–1^ and can be calculated by the Clausius–Clapeyron equation d*T*_t_/d*p* = Δ*V*_t_/Δ*S*_t_ (where Δ*V*_t_ and Δ*S*_t_ are the specific volume change and entropy change of the phase transition, respectively).^27,28^

In the specific case of the breathing-caloric MOF-508b, the situation is different, as the transition temperature can be shifted 80 K by applying only 25 bar, and the pressure dependence is not linear. This significantly larger modulation cannot be justified by Clausius–Clapeyron evaluating just the framework structural transition but must be addressed considering both framework and adsorbed gas thermodynamic parameters. For that reason, in this work, we have applied the very well-known thermodynamic osmotic ensemble model^29–31^ to calculate the transition temperature modulation with pressure, using isotherms extracted from the scientific literature^22^ further complemented with our high-pressure HP-TGA data (see Figure 2c). Table 1 compiles the thermodynamic parameters obtained from the isotherm Langmuir fitting of the adsorption branches and from the osmotic potential calculations (see details in Supporting Information). From these data, we have numerically solved the osmotic potential equation in the equilibrium state of the phase transitions, following the procedure reported elsewhere,^29–31^ to then calculate the *p*–*T* phase diagram from thermodynamic parameters.

**Table 1:** Thermodynamic Parameters for the Host Structural Transition and for the Host–Guest Adsorption Process Obtained from the Langmuir Fitting of the CO_2_ Adsorption Isotherms and from the Osmotic Potential Calculations

thermodynamic parameters for the host structural transition	
Δ*H*_host_ (np to ifp) = 25.4 kJ mol^–1^	Δ*S*_host_ (np to ifp) = 16.8 J mol^–1^ K^–1^
Δ*H*_host_ (ifp to lp) = 51.0 kJ mol^–1^	Δ*S*_host_ (ifp to lp) = 76.6 J mol^–1^ K^–1^

thermodynamic parameters for the host–guest adsorption process				
phase	*K*′ (molecules u.c.^–1^ bar^–1^)	Δ*H*_ads_ (kJ mol^–1^)	*A* (molecules u.c.^–1^)	*B* (molecules u.c.^–1^ K^–1^)
np	1.8 × 10^–6^	30.0	16.9	0.04
ifp	1.5 × 10^–5^	30.3	12.1	0.02
lp	2.2 × 10^–4^	24.9	56.4	0.13

As it can be observed in Figure 2d, the *p*–*T* boundaries of the np-ifp and ifp–lp transitions obtained by the osmotic ensemble model using the adsorption isotherms are in full agreement with those obtained by VT-DSC at different pressures upon the cooling process (equivalent to the adsorption process). It should also be noted that the adsorption enthalpy change values are in agreement with the literature.^32–35^

Accordingly, we can conclude that the transition temperature modulation with pressure (d*T*_t_/d*p*) is fully dependent on the thermodynamic parameters from both the MOF structural transitions (Δ*H*_host_, Δ*S*_host_) and from the MOF-CO_2_ sorption process dominated by the adsorption affinity (*K*, *K*′) and enthalpy changes (Δ*H*_ads_), as well as by the saturation uptake (*N*_max_, *a*, *b*). In turn, a proper control of all these thermodynamic parameters will allow one to rationally tune the d*T*_t_/d*p* values in future breathing-caloric materials. Furthermore, from these data, we can estimate the different contributions to the total enthalpy change for the np-phase to lp-phase transition as |Δ*H*_t_| = |(*N*_max_^lp^ Δ*H*_ads_^lp^) + Δ*H*_host_(np to lp)|, which exhibits values as large as |Δ*H*_t_| ∼ 82.2 J g^–1^ at 273 K under adsorption saturation (where *N*_max_^lp^ = 5.1 mol_(CO_2_)_ mol_(MOF)_^–1^).

### 2.2 Pressure-Assisted Thermal Energy Storage in the Breathing-Caloric MOF-508b

In what follows, we will focus on the potential of MOF-508b for thermal energy storage and release. In that context, the heat charging process will be associated with the endothermic transitions upon heating and imply the release of CO_2_. Meanwhile, the heat discharge will be related to the exothermic phase transitions upon cooling, where the CO_2_ adsorption occurs.

The thermal storage capacity of MOF-508b at different pressures (Figure 2e) can be calculated as energy density (*E*) according to eq 1:

1 $$E=\left\{\Delta H_{t}+\left[\int_{T_{i}}^{T_{f}}C_{p}\;dT\right]\right\}$$

where Δ*H*_t_ is the total enthalpy change of the two combined phase transitions and *C*_p_ is the specific heat capacity obtained independently from the phase transition and any CO_2_ gas adsorption, as described in the Experimental Section.

In that regard, on heating (h) and cooling (c) under isobaric conditions of 5 bar of CO_2_, MOF-508b exhibits values of Δ*H*_t(h)_ = 61.1 J g^–1^ and Δ*H*_t(c)_ = 83.2 J g^–1^, respectively (Figure 2a inset). These experimental values are in the same range of the theoretical values predicted by the osmotic ensemble model. It should be noted that the values of Δ*H*_t(c)_ (related to CO_2_ adsorption process) are noticeably larger than Δ*H*_t(h)_ (related to the CO_2_ desorption process). This phenomenon was also observed by Barbour et al., who also found significantly larger values for Δ*H*_t(0→p)_ (adsorption process) than for Δ*H*_t(p→0)_ (desorption process). This difference was attributed to potential guest trapping interactions,^23^ which might be affecting the desorption kinetics and the observable thermal energy. Moreover, the thermal hysteresis might also contribute to this aspect, where, upon heating, a slight amount of CO_2_ molecules is released before the phase transition (see Figure 1d) without being detected by our VT-DSC.

The here-observed thermal changes are maintained under pressures of up to 25 bar, showing a slight decrease down to Δ*H*_t(h)_ = 51.2 J g^–1^ and Δ*H*_t(c)_ = 71.9 J g^–1^. The pressure-dependence of the enthalpy change values can be associated with the equilibrium between external and internal CO_2_ molecules. At higher pressures (and higher CO_2_ external concentrations), MOF-508b will release a lower amount of CO_2_ molecules, leading to a corresponding decrease in the enthalpy change during the phase transition.

Meanwhile, the specific heat capacity increases from ∼0.4 J g^–1^ °C^–1^ to ∼1.2 J g^–1^ °C^–1^ in the temperature range from −30 to 120 °C (Figure 2e inset), values that are in agreement with those reported in the literature.^33,36^

Therefore, considering both contributions, the energy density of MOF-508b can be as large as ∼160 J g^–1^ (∼240 kJ L^–1^ considering a reported density of ∼1.5 g cm^–3^ for activated MOF-508b^37^) at 5 bar upon cooling from 90 °C down to −30 °C. This energy density is slightly decreased to ∼140 J g^–1^ (∼210 kJ L^–1^) upon heating, due to the difference observed between Δ*H*_t(c)_ and Δ*H*_t(h)_ (see Figure 2f). It should be noted that this energy density is normalized per MOF mass (and volume) and is in the range of that for commercial TES materials.^8^ For real technological applications, the required amount of CO_2_ must also be considered. Evaluating the CO_2_ adsorption isotherms at 5 bar, the MOF-508b exhibits a gas uptake of ∼6 mmol_(CO2)_ g_(MOF)_^–1^ (0.26 g_(CO_2_)_ g_(MOF)_^–1^) when decreasing the temperature from 65 °C down to 0 °C (Figure 2c). Therefore, the energy density under this conditions can be expressed as normalized per MOF mass, *E*_(MOF)_ ∼ 116.5 J g_(MOF)_^–1^, or as normalized per mass of MOF and adsorbed CO_2_, *E*_(MOF‑CO_2_)_ ∼ 92.5 J g_(MOF‑CO_2_)_^–1^ (or ∼120.3 kJ L^–1^ considering a density of ∼1.3 g cm^–3^ for CO_2_-filled MOF-508b^22^), which is still a significantly large value.

Furthermore, VT-DSC cycling studies over time confirm that the process is fully reversible for at least 14 h (Figure S9 of Supporting Information), in agreement with our VT-TGA cycling data (Figure S7 of Supporting Information).

### 2.3 Pressure Modulation of the Critical Temperatures for Heat Charging and Discharging

Very interestingly, the VT-DSC data presented here reveal that it is possible to modulate the energy-charging/discharging temperatures by setting different CO_2_ pressures (Figure 2a). On this basis, we identify three different charge/discharge thermal cycles with MOF-508b that could be adapted depending on technological and/or environmental needs. These thermal cycles present different CO_2_ conditions: (i) CO_2_ isobaric conditions during charge and discharge (*p*_charge_ = *p*_discharge_), (ii) CO_2_ pressures larger during the charge (*p*_charge_ > *p*_discharge_), and (iii) CO_2_ pressure larger during the discharge (*p*_charge_ < *p*_discharge_).

In the first case (*p*_charge_ = *p*_discharge_), by setting a constant pressure (for instance, 15 bar), the charge and discharge temperatures only rely on the thermal hysteresis of the material. Remarkably, these temperatures can be increased by setting a higher CO_2_ constant pressure (i.e., 25 bar) or decreased by setting a lower CO_2_ pressure (i.e., 5 bar) (see Figure 3a,b). In this example, the charge/discharge temperatures (measured at the peaks’ maxima) will decrease from 94 °C/63 °C (367 K/336 K) at 25 bar down to 35 °C/16 °C (308 K/289 K) at 5 bar. Nevertheless, the total energy will not be absorbed and/or released until the transitions are completed. Therefore, the actual thermal hysteresis will be even larger.

**Figure 3:**
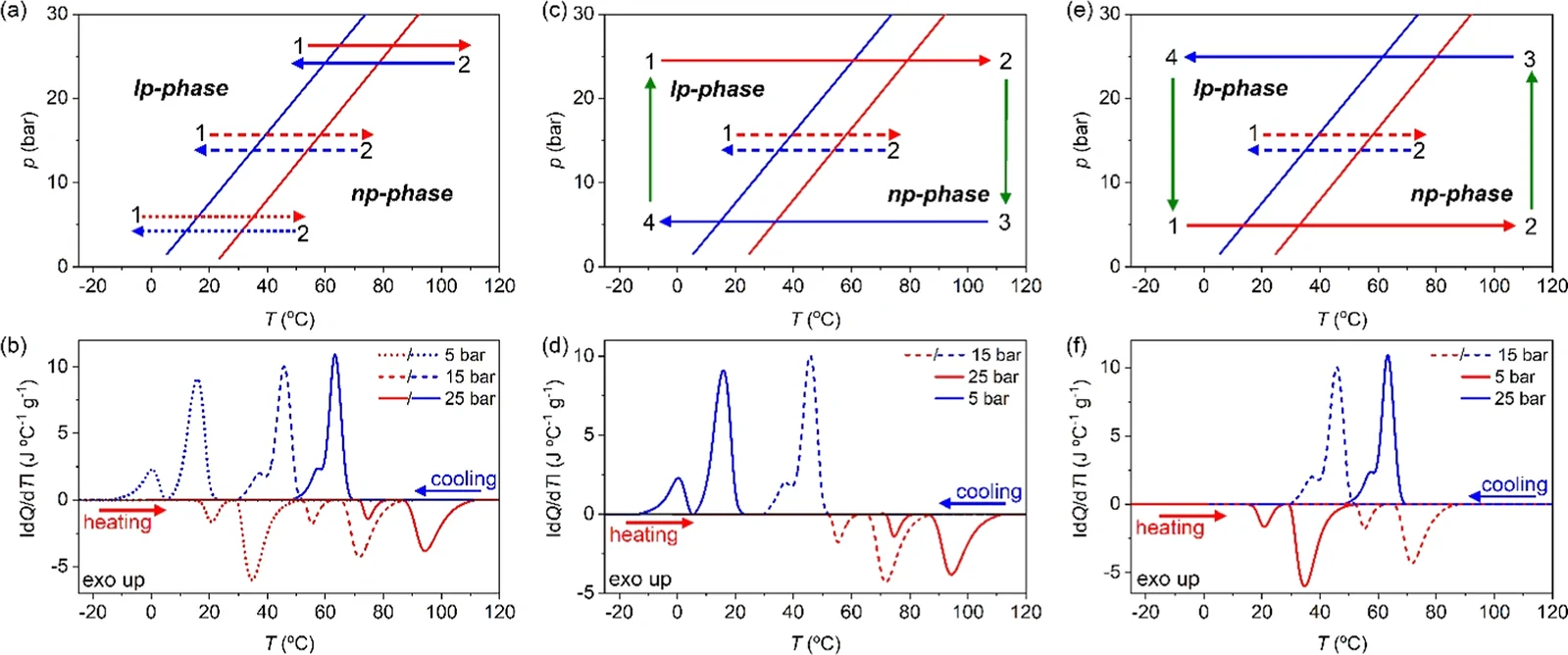
Simplified schematic *p*-*T* phase diagrams of MOF-508b showing the thermal cycles and associated VT-DSC for different CO_2_ pressure conditions: (a,b) isobaric CO_2_ conditions (*p*_charge_ = *p*_discharge_), (c,d) CO_2_ pressures larger during the charge (*p*_charge_ > *p*_discharge_), and (e,f) CO_2_ pressure larger during the discharge (*p*_charge_ < *p*_discharge_). Note: Solid lines represent the corresponding thermal cycles, and dashed lines represent alternative isobaric cycles for comparison. The thermal cycles can be performed in 2 steps (1 ↔ 2) for direct heating ↔ cooling at constant pressure or in 4 steps (1 → 2 → 3 → 4 → 1) for different pressure settings, where steps (1 → 2) and (3 → 4) indicate, respectively, the heating and cooling processes and steps (2 → 3) and (4 → 1) indicate the changes in the pressure setting.

These experimental results thus reveal that the same phase change material—MOF-508b—can be used for thermal energy storage, adapting to cold and heat environments and climates just by tuning the surrounding CO_2_ pressure.

In the second type of TES cycle (*p*_charge_ > *p*_discharge_), the thermal hysteresis can be increased by setting a given isobaric pressure during the energy-charge and a different isobaric pressure during the energy-discharge processes. A representative example of this cycle would consist of setting the CO_2_ isobaric pressure at 25 bar and then heating the material, which will exhibit an energy-charge temperature of 94 °C (367 K), see Figure 3c,d. Meanwhile, if we reset the isobaric pressure to 5 bar before cooling (without inducing any phase transition), the discharge temperature gets modified to 16 °C (289 K). Therefore, in this case, the thermal hysteresis is 78 °C (351 K), see Figure 3c,d. This value is significantly larger than the material’s original thermal hysteresis at 5 bar (*T*_charge_ – *T*_discharge_ ≈19 °C), thus demonstrating that it can be tuned and adapted to the required application.

With the third type of TES cycle (*p*_charge_ < *p*_discharge_), using the inverse strategy, the thermal hysteresis can be decreased and even reversed—a process not described so far in any other TES material. For example, for a starting isobaric condition of 5 bar, the charge temperature upon heating will be 35 °C (308 K). Meanwhile, by resetting the isobaric conditions at 25 bar before cooling down (without inducing any phase transition), the discharge temperature will be 63 °C (336 K). Therefore, under these conditions (represented in Figure 3e,f), the energy-discharge temperature is significantly larger than the energy-charge temperature. In turn, the thermal hysteresis has been reversed with a value of −28 °C (245 K). Figure S10 of the Supporting Information shows a continuous VT-DSC cycle over time for this third scenario, where the pressure is isothermally increased after the heating ramp (thermal energy storage process) for obtaining a subsequent cooling ramp (thermal energy release process) at a higher pressure: *p*_charge_ < *p*_discharge_. In this cycle, it can be effectively observed that the discharge temperature (*T*_discharge_ or *T*_t(c)_) is higher than the initial charge temperature (*T*_charge_ or *T*_t(h)_) in a continuous cycle. Furthermore, from this cycle, we can also obtain the thermal energy contribution of the CO_2_ during the isothermal pressure switching, which exhibits a value of ∼6.0 kJ kg^–1^ (normalized per MOF mass). This relatively small exothermic process is related to the pressure increase from 5 to 25 bar for the CO_2_ inside the DSC sample holder (∼190 μL), and its impact could be further minimized for real applications through adequate technological designs in the future.

Therefore, these results clearly demonstrated that a gas-responsive MOF enables the storage of heat at a given temperature and its subsequent release at much higher temperatures (instead of at lower temperatures as in traditional PCMs), overcoming one of the major limitations of TES based on PCMs, such as unalterable phase-transition temperatures.^13,14^ This temperature-reversal capability provides significant thermal management flexibility, which opens the door to the design of adaptive energy storage systems that can respond to variable operational conditions without changing the storage material.

In summary, these findings significantly increase the versatility of breathing-caloric materials. They could enable the design of multipurpose devices not only for conventional pressure cycling thermal technologies (fridges, air conditioners, heat pumps) but also for innovative pressure-assisted temperature-triggered thermal energy storage systems. In these alternative TES systems, the charge/discharge temperatures could be easily modulated by tuning the materials’ surrounding isobaric conditions, so they can adapt to different ambient temperatures (diurnal or seasonal), as well as to diverse storage needs (from cold storage to medium–high-temperature applications), see Figure 4.

**Figure 4:**
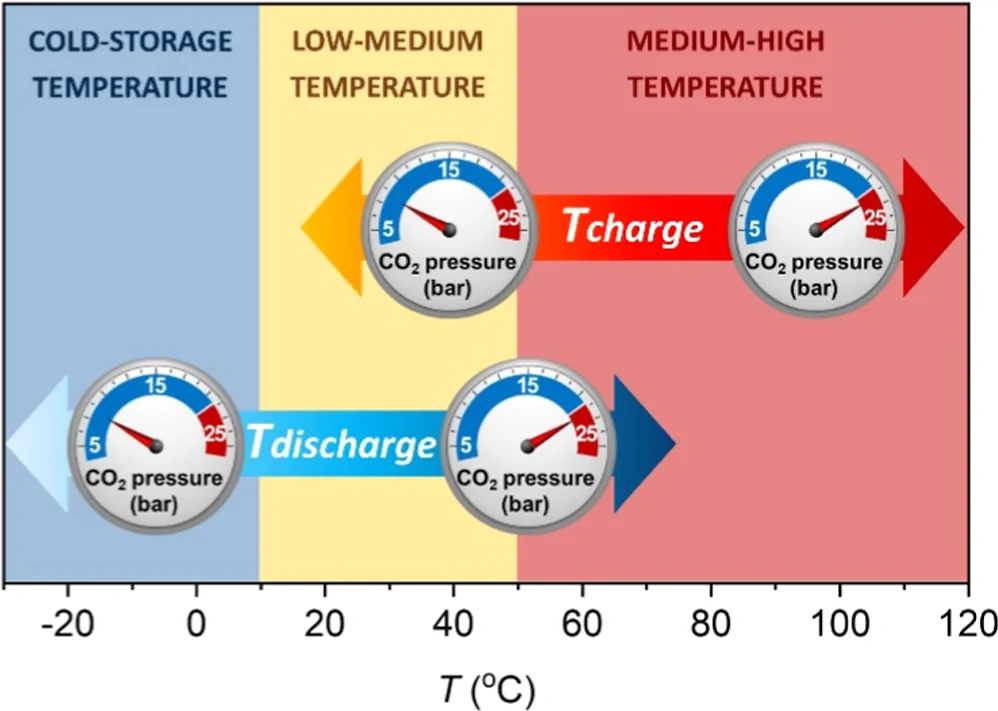
Illustration showing how the thermal energy charge and discharge temperatures (*T*_charge_, *T*_discharge_) can be easily tuned and even reversed (*T*_charge_ < *T*_discharge_) just by setting different CO_2_ isobaric conditions, where the thermal energy store and release can be adapted to the desired operational temperature ranges.

## 3 Conclusion

In this work, we reveal innovative pressure-assisted thermal energy storage mechanisms for flexible MOFs, based on tunable isobaric conditions and thermally induced breathing transitions, as illustrated by the case of MOF-508b. In this study, we combined VT-DSC studies with VT-TGA at different isobaric CO_2_ pressures. This latter is a technique scarcely used to evaluate MOFs but that can be very useful to study sorption thermal energy storage, as here demonstrated. Furthermore, we apply the thermodynamic osmotic ensemble model to calculate the pressure responsiveness of MOF-508b as well as the *p*–*T* phase diagram, which also reveals to be a very powerful tool to predict phase diagrams and thermodynamic parameters of interest for breathing-caloric and pressure-assisted thermal energy storage properties.

The here-presented results show that MOF-508b displays an energy storage density similar to commercial TES materials. In addition, and in contrast with conventional TES materials, the heat charge/discharge temperatures (*T*_charge_/*T*_discharge_) in MOF-508b can be modulated in the range of −30 to 120 °C (243–393 K) just by setting different isobaric CO_2_ conditions, even using a pressure range as narrow as 5–25 bar. Moreover, its thermal hysteresis (|*T*_charge_ – *T*_discharge_|) can be easily increased and decreased, and even reversed, just by setting different pressures in the charge and discharge process. In fact, by reversing it, it is possible to set an energy-discharge temperature that is higher than the energy-charge temperature (*T*_charge_ < *T*_discharge_).

These findings demonstrate the exceptional versatility of gas-responsive MOFs and establish a new paradigm for their application in adaptive thermal energy storage. We anticipate that the observed tunable thermal behavior is not unique to MOF-508b but represents a phenomenon that could be applicable to other MOFs exhibiting similar structural flexibility and gas-responsive characteristics. This opens new avenues for material discovery, as we envision that many switchable framework materials—and potentially other stimuli-responsive systems—could exhibit analogous tunable properties. We therefore encourage the community to explore this largely untapped potential, which could significantly expand the library of materials available for adaptive TES applications.

## 4 Experimental Section

### 4.1 Synthesis

The MOF-508b material was obtained by adapting the method reported by M. Bonneau et al.^37^ Terephthalic acid [H_2_bdc] (Merck 98%), 4,4′-bipyridine [bpy] (Merck 98%), Zn­(NO_3_)_2_·6H_2_O (Merck 99%), *N*,*N*-dimethylformamide [DMF] (Fischer Scientific ≥99.8%), absolute ethanol [EtOH] (Scharlab), and hexane (Labkem) were used without further purification. A mixture of H_2_bdc (0.498 g, 3 mmol) in DMF (20 mL), Zn­(NO_3_)_2_·6H_2_O (0.892 g, 3 mmol) in EtOH (15 mL), and bpy (0.234 g, 1.5 mmol) in DMF (7.5 mL) was stirred at 373 K during 24 h using a reflux synthesis setup. A light-yellow precipitate was collected by vacuum filtration, washed with hexane for 24 h under stirring, and dried under vacuum at *T* = 393 K for 24 h to finally obtain the desired MOF-508b compound.

### 4.2 Powder X-ray Diffraction

The obtained material was characterized by powder X-ray diffraction (PXRD) using a Siemens D-5000 diffractometer with Cu­(Kα) radiation at room temperature. The obtained patterns were compared with those simulated from reported single-crystal XRD,^22^ confirming that the material was obtained as a single-phase polycrystalline powder (see Figure S1 of Supporting Information).

The thermal evolution of the MOF-508b structure under different CO_2_ isobaric conditions was studied by synchrotron powder X-ray diffraction (SPXRD) using a wavelength of λ = 0.71073 Å. These studies were carried out at the BM01 Swiss Norwegian Beamline (SNBL) of the European Synchrotron Radiation Facility (ESRF),^38^ and the data is published under the digital object identifiers doi.org/10.15151/ESRF-ES-819989408 and doi.org/10.15151/ESRF-ES-819989408. For those experiments, 0.5 mm diameter quartz capillaries were filled with MOF-508b powder and measured in situ at isobaric conditions of 8 bar under a CO_2_ atmosphere between −33 and 107 °C (240 and 380 K). The temperature was controlled with an Oxford Cryostream 700+, and diffraction data were collected with a Pilatus 2 M detector. The recorded 2D patterns were integrated into a 1D powder profile and fitted using the Le Bail method (Figures S4–S6 of Supporting Information). The diffraction patterns were refined using the GSAS-II software.^39^

### 4.3 Nuclear Magnetic Resonance Spectroscopy

^1^H NMR and ^13^C NMR were used to confirm the bdc/bpy ratio and absence of impurities in the obtained MOF-508b compound using Bruker Avance 300 MHz equipment. Chemical shifts (δ in ppm) were determined according to the residual signal of the solvent. The samples were dissolved in a mixture of deuterated DMSO-*d*_6_ (∼0.6 mL) and deuterated trifluoroacetic acid (∼14 μL) following reported conditions.^37^ The bdc/bpy ratio was found to be 2:1, calculated as the ratio of the ^1^H signals of the respective building blocks, and no additional bdc and/or further organic impurities were observed (see Figure S2 of Supporting Information).

### 4.4 Isothermal Adsorption Analysis

The N_2_ sorption isotherm was measured at 77 K using an ASAP 2020 Micromeritics instrument. The sample was previously degassed at 393 K under vacuum for 24 h. For comparison purposes, we calculate the BET (680 m^2^ g^–1^) and Langmuir (772 m^2^ g^–1^) surface areas from the N_2_ adsorption isotherm (see Figure S11 of Supporting Information), which are in full agreement with the literature.^19,24^

### 4.5 Thermogravimetric Analysis

High-pressure thermogravimetric analysis was performed in a TA Instrument Discovery HP-TGA 7500. Samples of around 15 mg were analyzed under different pressure and temperature conditions. Reversible cycles were performed from 65 to 120 °C (338 to 393 K) at a rate of 5 °C min^–1^ at isobaric conditions of 20 bar of CO_2_. Thermal decomposition was monitored from 65 to 900 °C (338 to 1173 K) at a rate of 5 °C min^–1^ at three different isobaric conditions (2, 10, and 20 bar). The buoyancy effects were considered and corrected following the manufacturer protocol.

### 4.6 Differential Scanning Calorimetry

The pressure-assisted thermal energy storage was evaluated by variable-temperature differential scanning calorimetry (VT-DSC) from −30 to 120 °C (243 to 393 K) using heating and cooling rates of 1.2 °C min^–1^ and under different CO_2_ isobaric conditions from 5 to 25 bar in a Setaram microcalvet high-pressure differential scanning calorimeter. In all experiments, approximately 5 mg aliquots of powdered samples were used. The baseline was corrected by using empty pans as blank samples under the same conditions. The thermal changes were calculated by integrating the heat flow, taking into account the correction described above.

The specific heat capacity (*C*_p_) was obtained under variable temperature and at ambient pressure by the classic ASTM E1269 standard test method, where the heat flow of MOF-508b was compared to that of a sapphire reference material according to eq 2:^40^

2 $$C_{p}=C_{ps}\frac{D_{m}m_{m}}{D_{s}m_{s}}$$

where *C*_*p*_ is the heat capacity of MOF-508b, *D*_m_ is the vertical displacement between the MOF-508b heat flow curve and the baseline, *C*_*p*s_ is the tabulated heat capacity for the sapphire reference, *D*_s_ is the vertical displacement between the experimental sapphire heat flow curve and the baseline, and *m*_m_ and *m*_s_ are the masses of MOF-508b and sapphire, respectively.

The heat capacity experiments were performed in a temperature range of −30 to 120 °C (243 to 393 K) using a temperature rate of 5 °C min^–1^ and under a nitrogen atmosphere to avoid any contribution from the phase transitions and from any CO_2_ gas adsorption.

## Supplementary Material



## Notes

Additional thermodynamic and calculations details and additional structural, thermal, and sorption data (PDF)

## Abbreviations

TES: thermal energy storage
PCMs: phase change materials
MOFs: metal–organic frameworks
SPXRD: synchrotron powder X-ray diffraction
TGA: thermogravimetric analysis
DSC: differential scanning calorimetry
PSA: pressure swing adsorption
TSA: temperature swing adsorption
VT: variable temperature
VP: variable pressure
lp-phase: large-pore phase
ifp-phase: induced-fit pore phase
np-phase: narrow-pore phase
