# Supplementary material for: Gas–Responsive Metal–Organic Frameworks for Adaptive Thermal Energy Storage with Tunable Charge–Discharge Temperatures
Source: J Am Chem Soc. 2026 Jul 6;148(35):37779–88. doi: 10.1021/jacs.6c07093 (PMC13564443; doi:10.1021/jacs.6c07093)
Supplement: Supplementary file 1 [file ja6c07093_si_001.pdf]

Supporting Information

Gas-Responsive Metal-Organic Frameworks for Adaptive Thermal Energy Storage with Tunable Charge-Discharge Temperatures

María Gelpi[a], David González-Novo[a,b], Lorena Alonso-Marañón[a], Javier García-Ben[a], Álvaro Baaliña[b], Sonia Zaragoza[c], Julian Walker[d], Charles James McMonagle[e], Socorro Castro-García[a], María Antonia Señarís-Rodríguez[a], Manuel Sánchez-Andújar[a], and Juan Manuel Bermúdez-García[a*]*

M. Gelpi, D. González-Novo, L. Alonso-Marañón, J. García-Ben, S. Castro-García, M. A. Señarís-Rodríguez, M. Sánchez-Andújar, J. M. Bermúdez-García

CICA – Interdisciplinar Center of Chemistry and Biology, Department of Chemistry, Faculty of Sciences, Universidade da Coruna, Campus de Elviña 15071 A Coruna, Spain.

E-mail: m.andujar@udc.es, j.bermudez@udc.es

D. González-Novo, Á. Baaliña

Energy Engineering Research Group, University Institute of Maritime Studies, Nautical Sciences and Marine Engineering Department, Escuela Técnica Superior de Náutica y Máquinas (ETSNM), Universidade da Coruna, Paseo de Ronda 51, 15011 A Coruna, Spain

S. Zaragoza

Universidade da Coruna (UDC), Ferrol Industrial Campus, CITENI, 15403 Ferrol, A Coruna, Spain.

J. Walker

Department of Materials Science and Engineering, Norwegian University of Science and Technology, 7491 Trondheim, Norway.

C. J. McMonagle

Swiss–Norwegian Beamlines, European Synchrotron Radiation Facility, Grenoble, 38043, France.

1. Thermodynamic osmotic ensemble details

In order to analyze the significantly large modulation of the transition temperature with pressure, we apply the thermodynamic osmotic ensemble[1–3] model using the CO_2 adsorption isotherms extracted from the scientific literature[4] and complemented with our HP-TGA data (see Figure 2c of the main manuscript).

In the last decades, the osmotic ensemble model has been demonstrated to be a very useful tool to describe structural transitions induced by fluid adsorption in metal-organic frameworks (MOFs).[1–3]

This model allows to calculate the *p*-*T* phase diagram of a given flexible MOF, and – in turn – also the transition temperature modulation with pressure considering basic thermodynamic parameters such as the Henry's adsorption affinity constant (K), the saturation adsorbed gas molecules (N_{max}), the adsorption enthalpy change (ΔH_{ads}), the phase transition enthalpy change (ΔH_{host}) and the phase transition entropy change (ΔS_{host}).[1–3]

From the CO_2 adsorption isotherms (Figure 2c of the main manuscript), we can obtain the values of K and N_{max} for each i-phase (i = np, ifp or lp) by fitting the curves to the Langmuir equation S1, according to the literature:[1–3]

$$N_{ads}^{(i)} = \frac{K_{(i)}P}{1+\frac{K_{(i)}P}{N_{max}^{(i)}}} \qquad \text{(Eq. S1)}$$

From these data, the adsorption enthalpy change ΔH_{ads} can be obtained from the Henry's constant fitting according to equation S2:[1–3]

$$K_{(i)} = K'_{(i)} e^{\left(-\Delta H_{ads}/RT\right)} \qquad \text{(Eq. S2)}$$

Accordingly, the osmotic potential difference between two different i-phases can be calculated following the equation S3 as reported elsewhere:[1–3]

$$\Delta\Omega_{os} = \Delta F_{host} + P\Delta V - RT[N_{max}^{(2)} \ln\left(1 + \frac{K_{(2)}P}{N_{max}^{(2)}}\right) - N_{max}^{1} \ln\left(1 + \frac{K_{(1)}P}{N_{max}^{(1)}}\right)] \qquad \text{(Eq. S3)}$$

In Eq. S3, ΔF_{host} is defined as the Helmholtz's free energy and $P\Delta V$ is the product of the transition pressure and the transition volume change. This latter value is much smaller than the rest of the contributions, so it is generally neglected in this equation for MOFs in general,[1–3] and for MOF-508b in particular[5]. Accordingly, the Helmholtz's free energy can be approximated to $\Delta F_{host} \sim \Delta G_{host} = (\Delta H_{host} - T\Delta S_{host})$, where ΔG_{host}, ΔH_{host} and ΔS_{host} are the Gibbs free energy change, the enthalpy change and the entropy change of the MOF phase transition, respectively.[1–3,5] Furthermore, the temperature dependence of K_i and N^{i}_{max} must be considered where $K_{(i)} = K'_{(i)}\, e^{(-\Delta Hads/RT)}$ and $N^{(i)}_{max} = a - bT$ (a and b are linear fitting values for $N^{(i)}_{max}$ versus T).

At a given phase transition, the osmotic potential of both involved phases are in equilibrium $\Omega^{(1)}_{os} = \Omega^{(2)}_{os}$ ($\Delta\Omega_{os} = 0$), and the value of ΔF_{host} can calculated for the T and P values of the measured adsorption isotherms. Once the value of ΔF_{host} is calculated, we can numerically solve the osmotic potential difference equation (Eq. S3) to model the complete p-T phase diagram where the different phases are in equilibrium.

2. Gravimetric and volumetric energy density calculations details

The thermal energy density calculations were performed using variable temperature differential scanning calorimetry (VT-DSC) data obtained as described in the experimental section of the main manuscript.

The thermal energy density, E, was calculated from the contributions of the total enthalpy change of the two consecutive thermally driven transitions of MOF-508b (ΔH_t) under different isobaric conditions and the specific heat capacity of MOF-508b (Cp) obtained under variable-temperature at ambient pressure, according to equation S4:

$$E = \left\{\Delta H_t + \left[\int_{T_i}^{T_f} Cp\, dT\right]\right\} \qquad \text{(Eq. S4)}$$

The total enthalpy change (ΔH_t) of the two consecutive transitions was obtained by integrating the peaks following equation S5:

$$\Delta H_t = \int_{T(onset)}^{T(offset)} \frac{dQ}{dT} dT \qquad \text{(Eq. S5)}$$

where the dQ/dT is the heat flow obtained by VT-DSC, $T_{(onset)}$ and $T_{(offset)}$ are the peak integration limits –the initial and final temperatures of the thermal transition selected where the curve deviates from the baseline–.

In this work, similarly to thermomaterials with pressure-induced barocaloric effects, the specific heat capacity (*Cp*) of MOF-508b is considered independent of pressure and similar upon heating and cooling.[6,7] In that line, for the energy density calculations we use the *Cp* calculated at ambient pressure upon heating under nitrogen atmosphere to avoid any contribution from the phase transitions and from any CO_2 gas adsorption. In turn, the C*p* was obtained by the ASTM E1269 standard test method using a sapphire reference material following equation S6:[8]

$$Cp = Cp_s \frac{D_m m_m}{D_s m_s} \qquad \text{(Eq. S6)}$$

where *Cp* is MOF heat capacity, D_m is the vertical displacement of the MOF heat flow curve from the baseline, Cp_s is the sapphire tabulated heat capacity, D_s is the vertical displacement of the experimental sapphire heat flow curve from the baseline, and m_m and m_s are the masses of MOF-508b and sapphire, respectively.

From these ΔH_t and C*p* data, we calculate the gravimetric energy density (normalized per MOF mass in units of $kJ\ kg^{-1}_{(MOF)}$), and also the volumetric energy density (normalized per MOF volume in units of $kJ\ L^{-1}_{(MOF)}$ considering the reported density of 1.5 g cm^{-3} for activated MOF-508b[5]). In the same line, we also normalize the thermogravimetric energy density by mass of MOF+CO_2 as obtained from the adsorption isotherms (Figure 2c of the main manuscript), as well as we normalize the volumetric energy density considering the reported density of 1.3 g cm^{-3} for the CO_2-filled MOF-508b.[4]

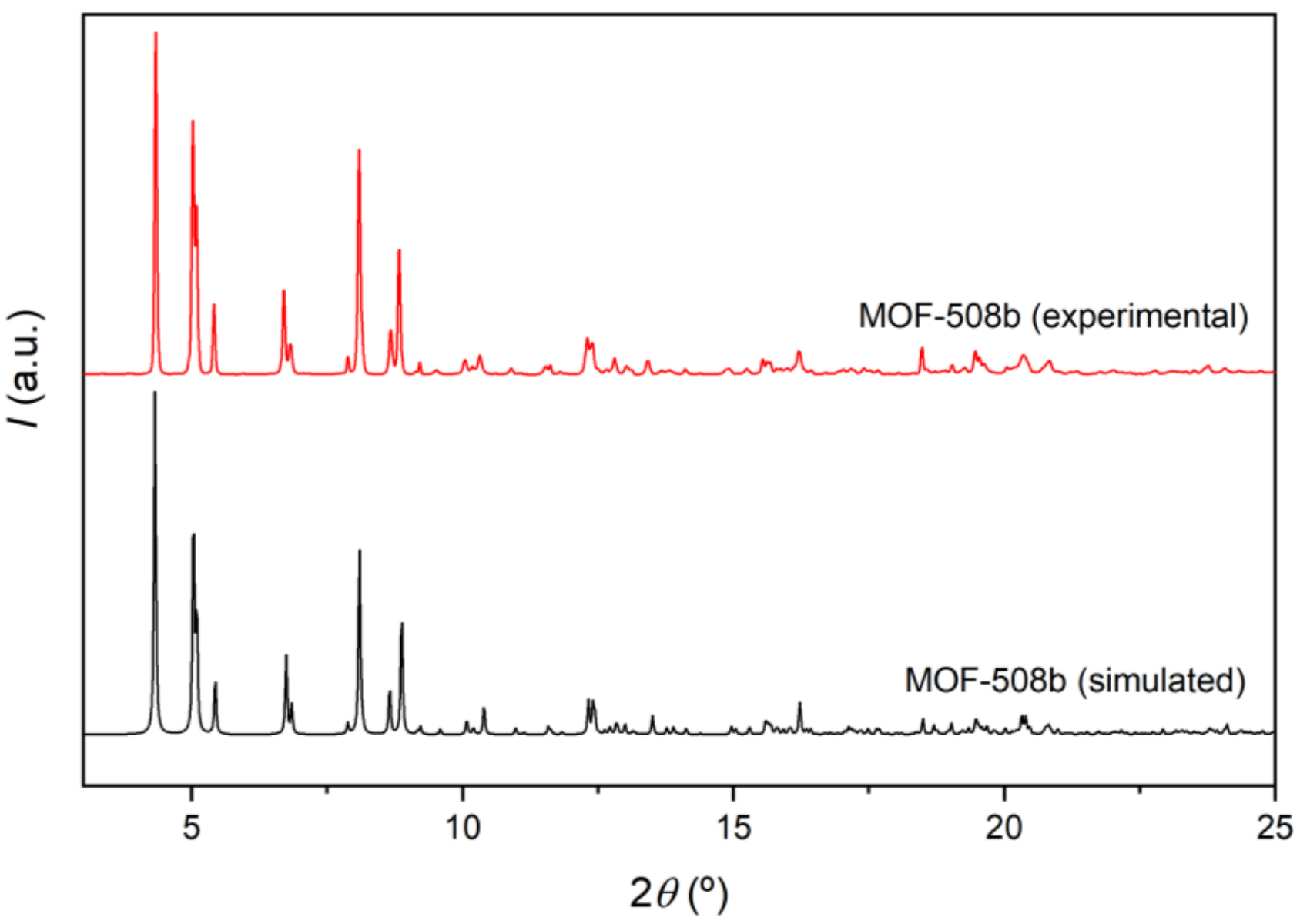

Figure S1. Powder XRD patterns of the here obtained MOF-508b compared with XRD patterns simulated from CIF files from reference [4].

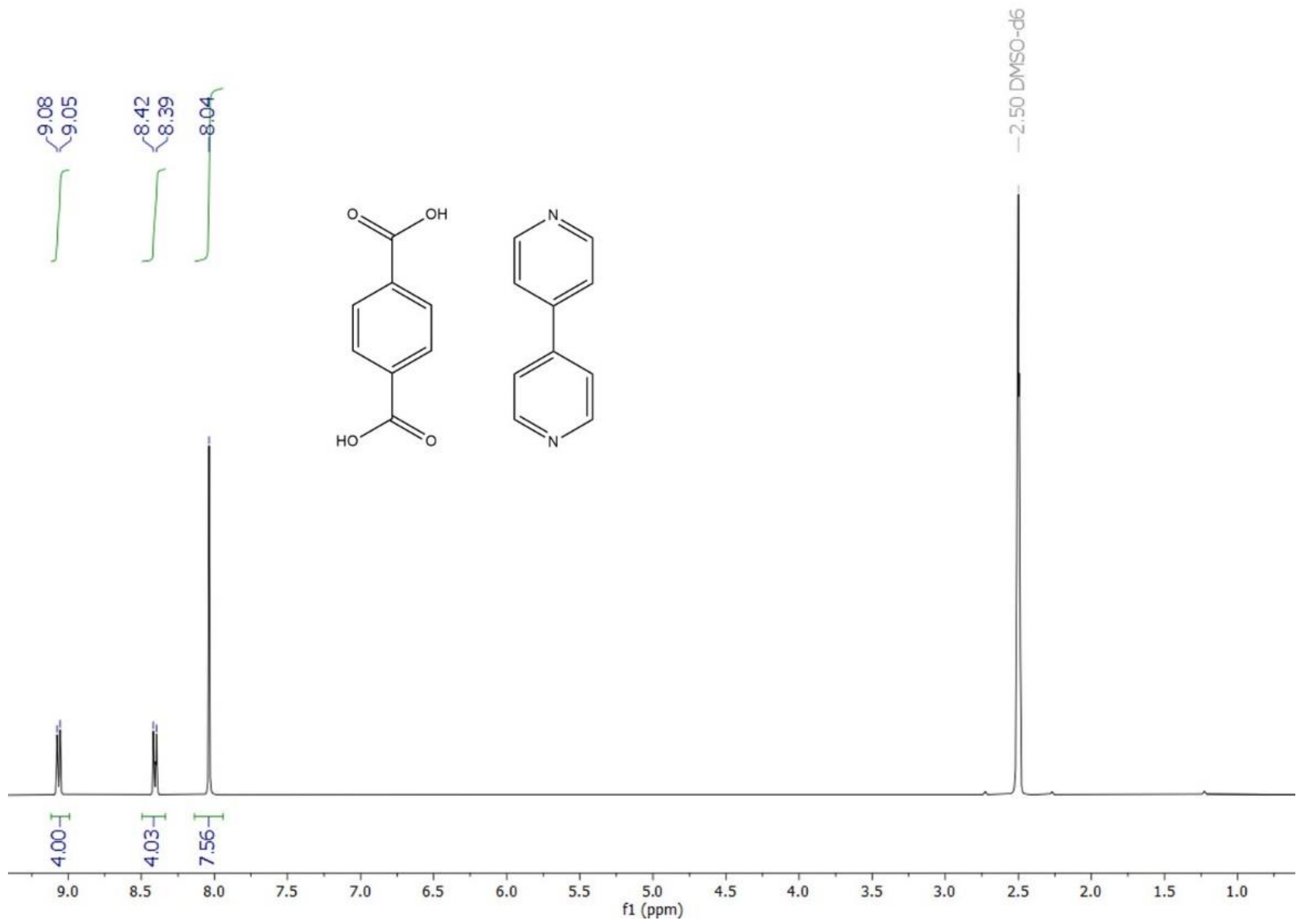

Figure S2. ^{1}H-NMR spectra for MOF-508b ($[Zn_2(bpy)(bdc)_2]$).

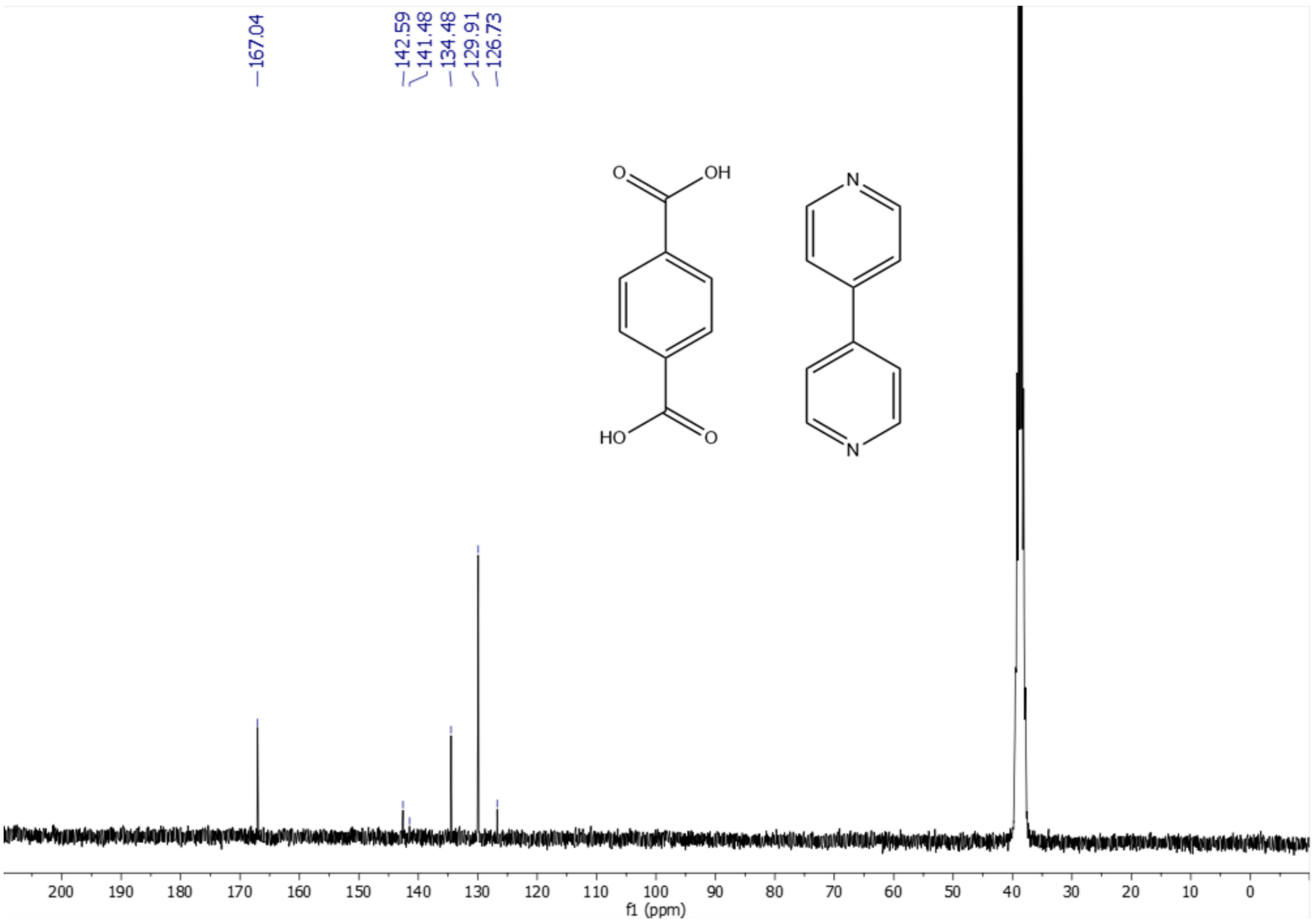

Figure S3. ^{13}C NMR spectra for MOF-508b ($[Zn_2(bpy)(bdc)_2]$)

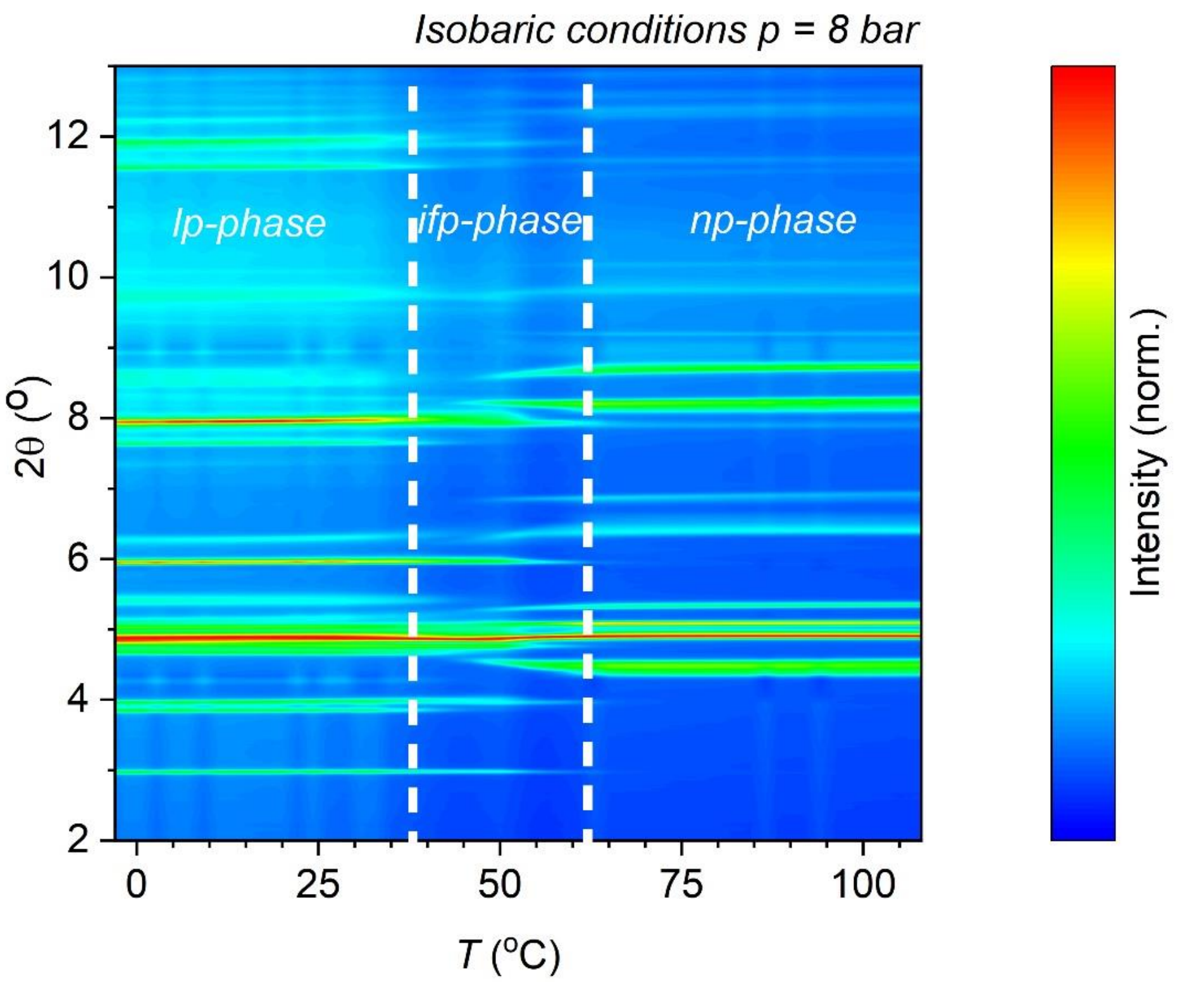

Figure S4. Representation of 2θ versus temperature of the synchrotron powder X-ray diffraction patterns under CO_2 isobaric conditions of 8 bar where the intensity is colour coded from higher (red) to lower (blue). Note: due to coexistance of phases in a certain temperature interval, the criteria used to indicate transition temperatures (marked as dashed lines) is the temperature above which the presence of lp-phase and the ifp-phase, respectively, is negligible.

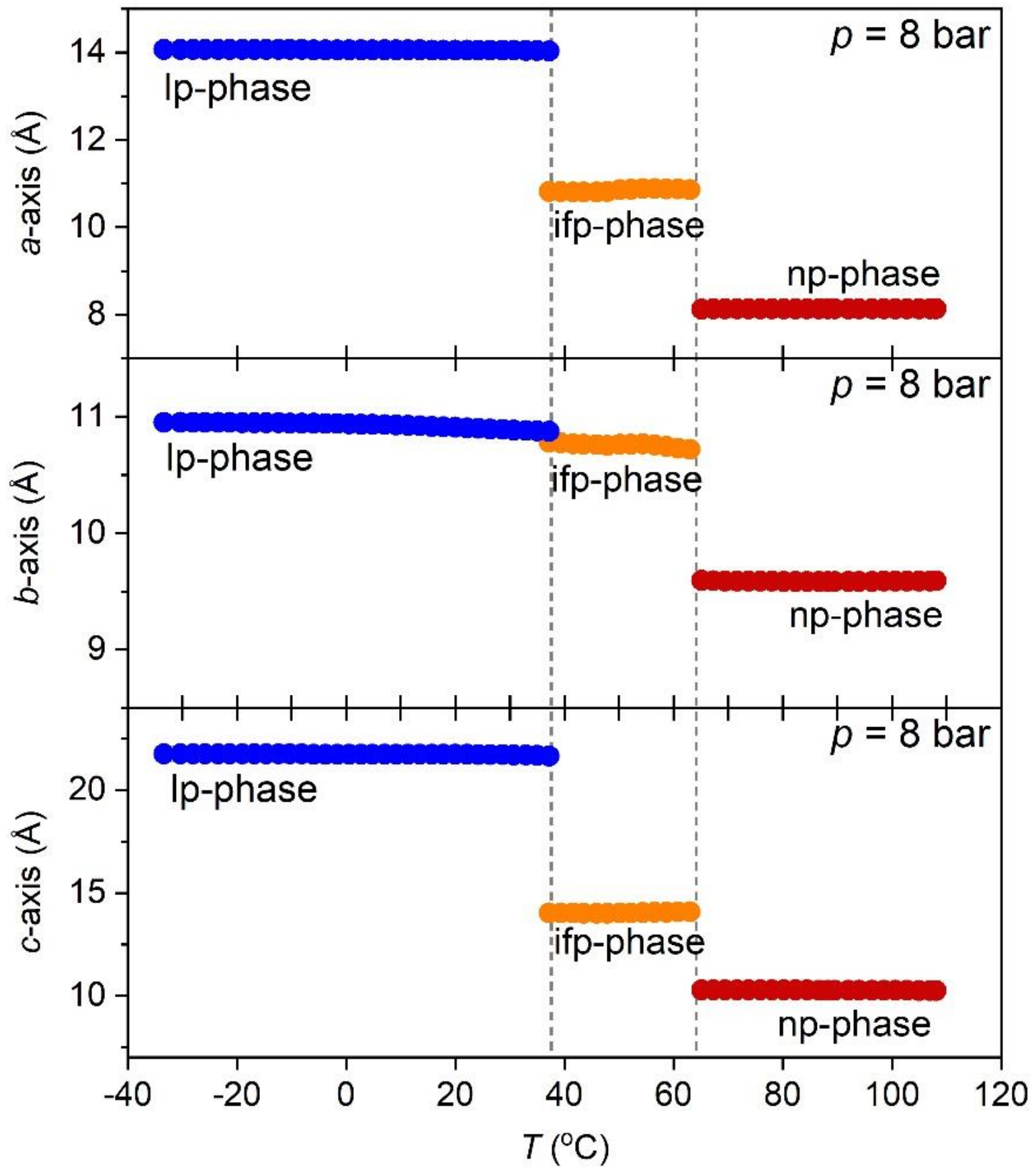

Figure S5. Evolution of the unit cell parameters (*a*-, *b*- and *c*-axis) upon heating under the isobaric CO_2 pressure of 8 bar, as obtained by SPXRD. Note: due to coexistance of phases in a certain temperature interval, the criteria used to indicate transition temperatures (marked as dashed lines) is the temperature above which the presence of lp-phase and the ifp-phase, respectively, is negligible.

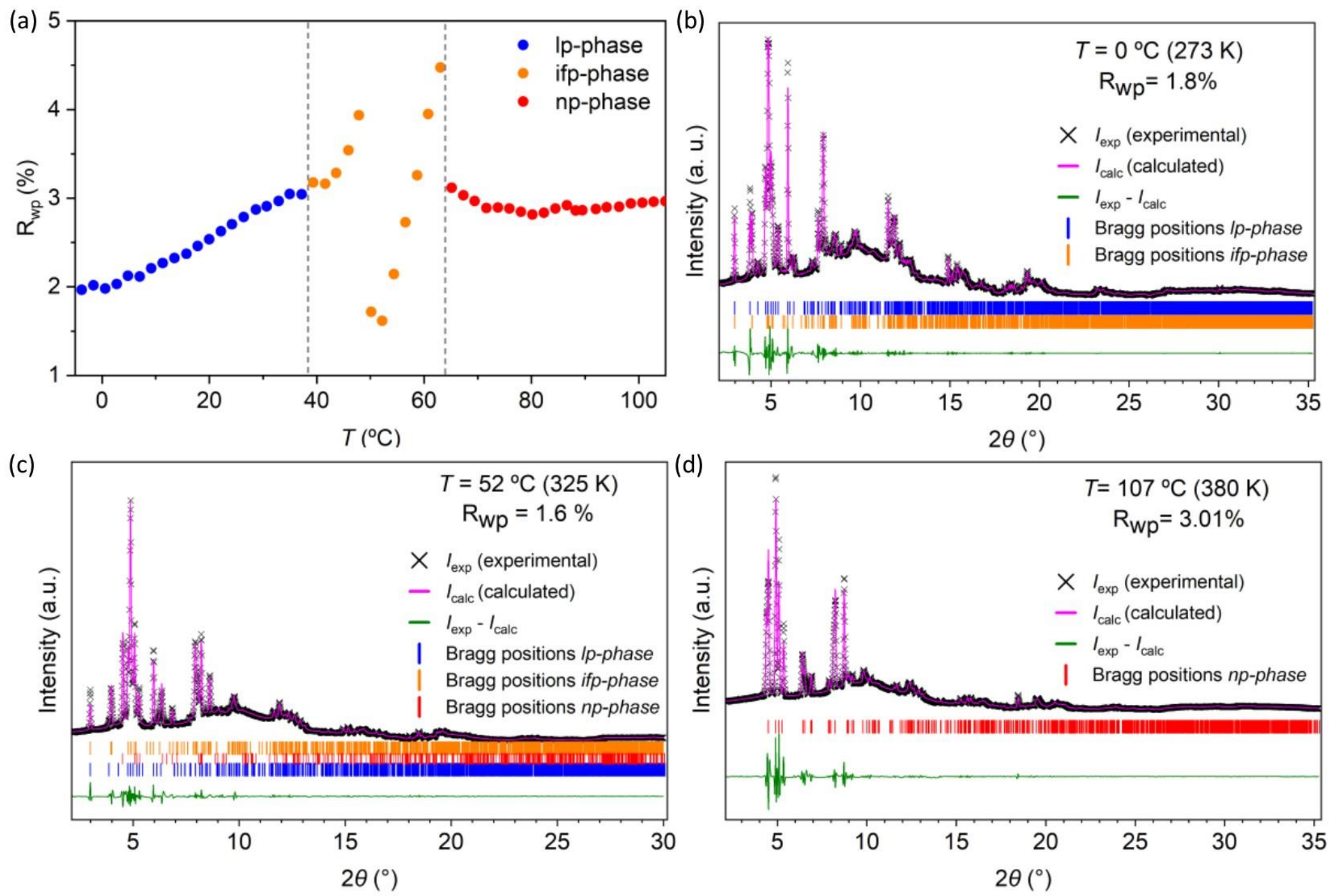

Figure S6. (a) Statistical R_{wp} for the Le Bail fittings at different temperatures, indicating the majoritary phase in colour code: blue = lp-phase, orange = ifp-phase, red = np-phase. (b-d) Representative Le Bail fittings at 0ºC (273 K), 52 ºC (325 K) and 107 ºC (380 K) showing phase coexistences.

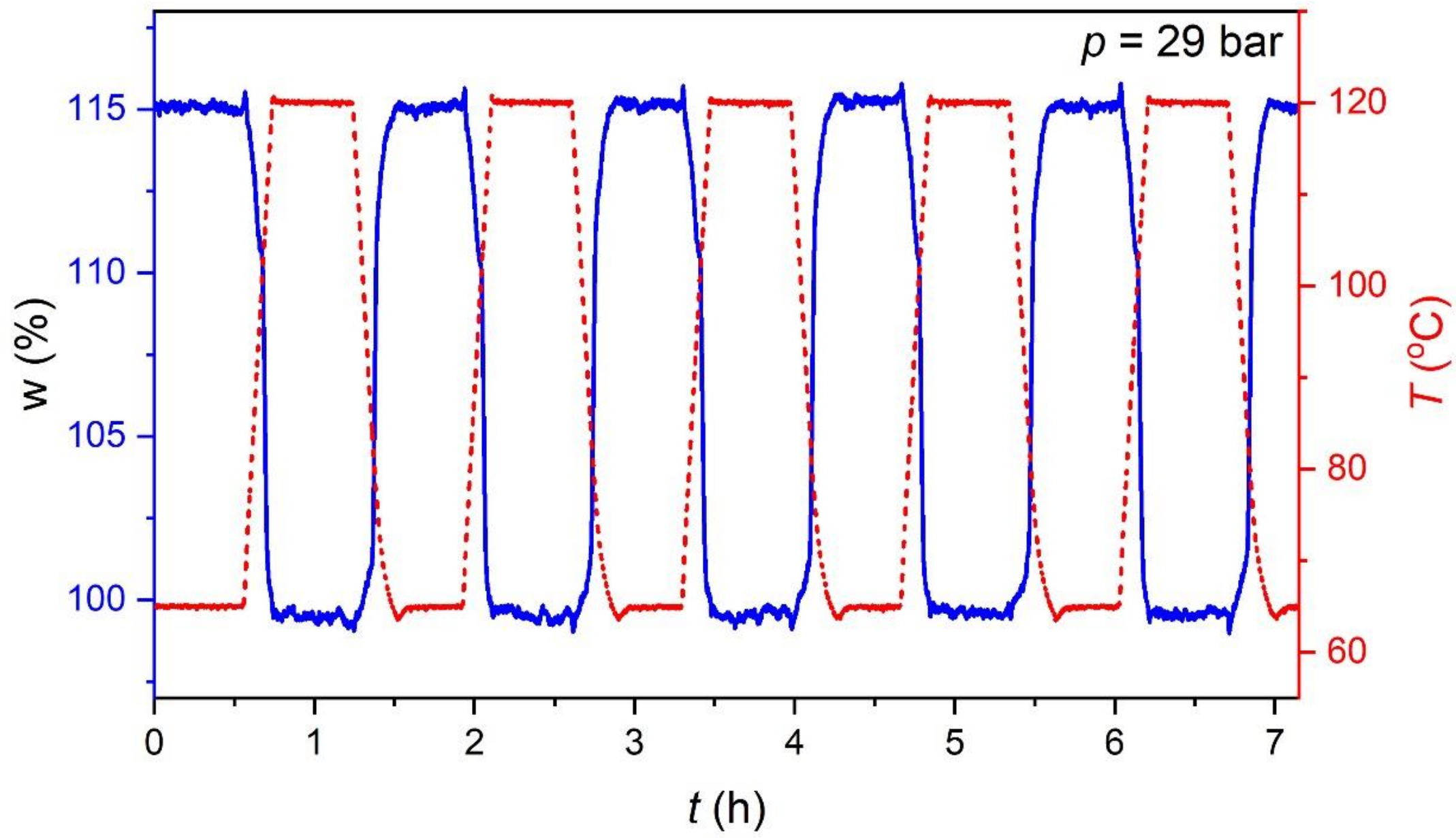

Figure S7. Cycling VT-TGA curves over time at 29 bar from 65 ºC to 120 ºC.

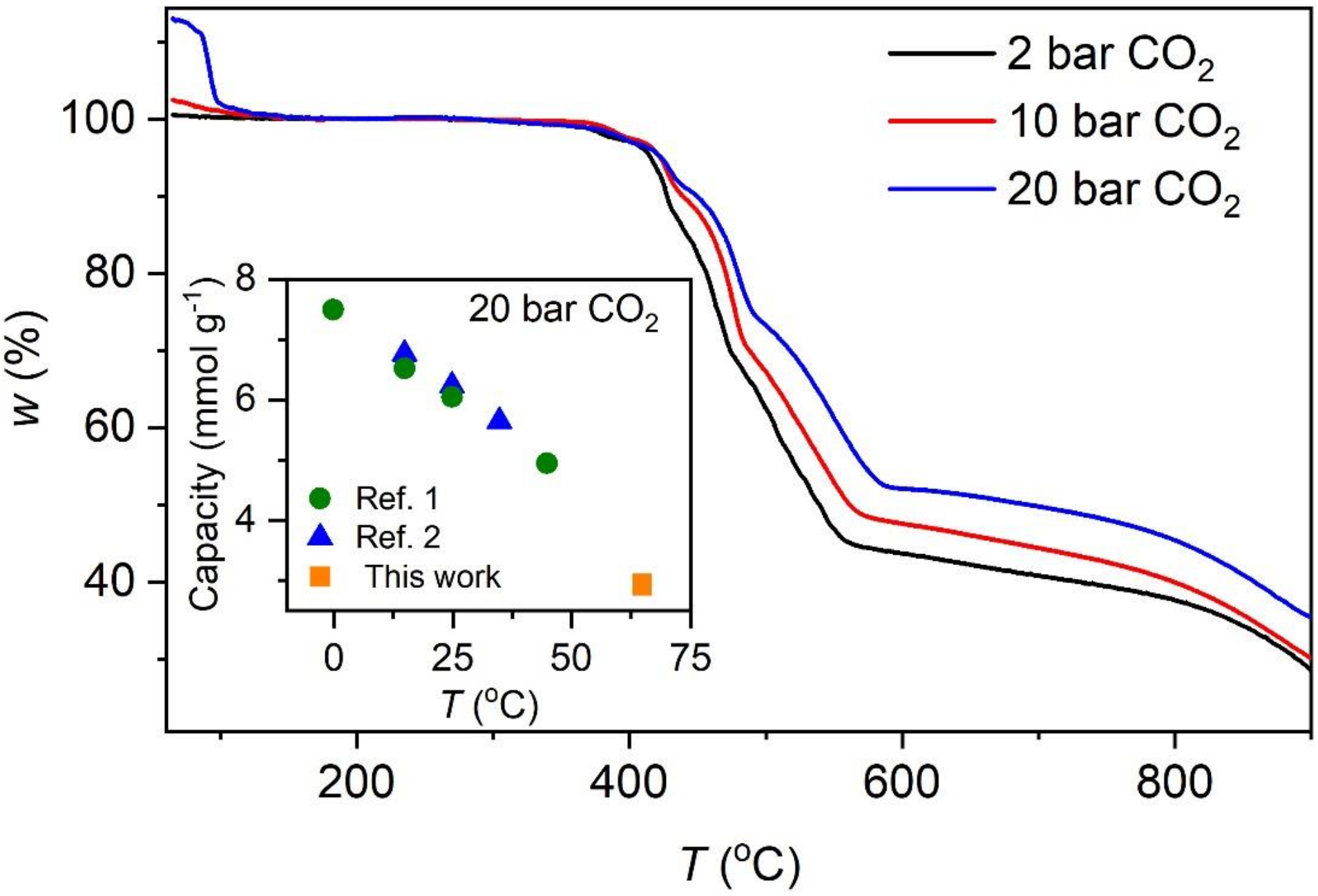

Figure S8. Thermal decomposition curves at different CO_2 isobaric conditions. Inset: Maximum adsorption capacity at 20 bar of CO_2 for different isothermal conditions (0-65 ºC). Inset data obtained from references[4,9].

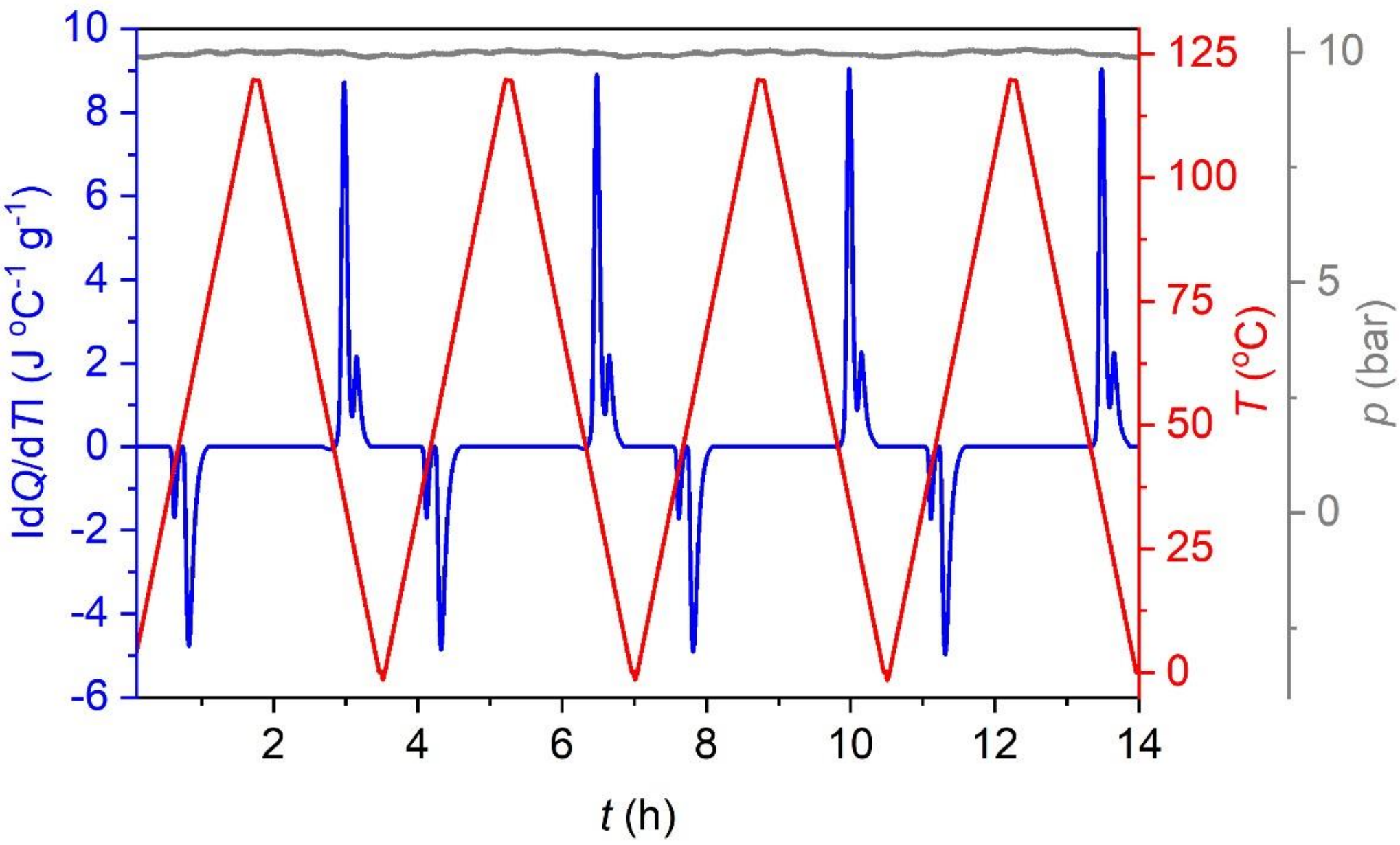

Figure S9. Cycling VT-DSC curves over time at 10 bar from 0 °C to 120 °C

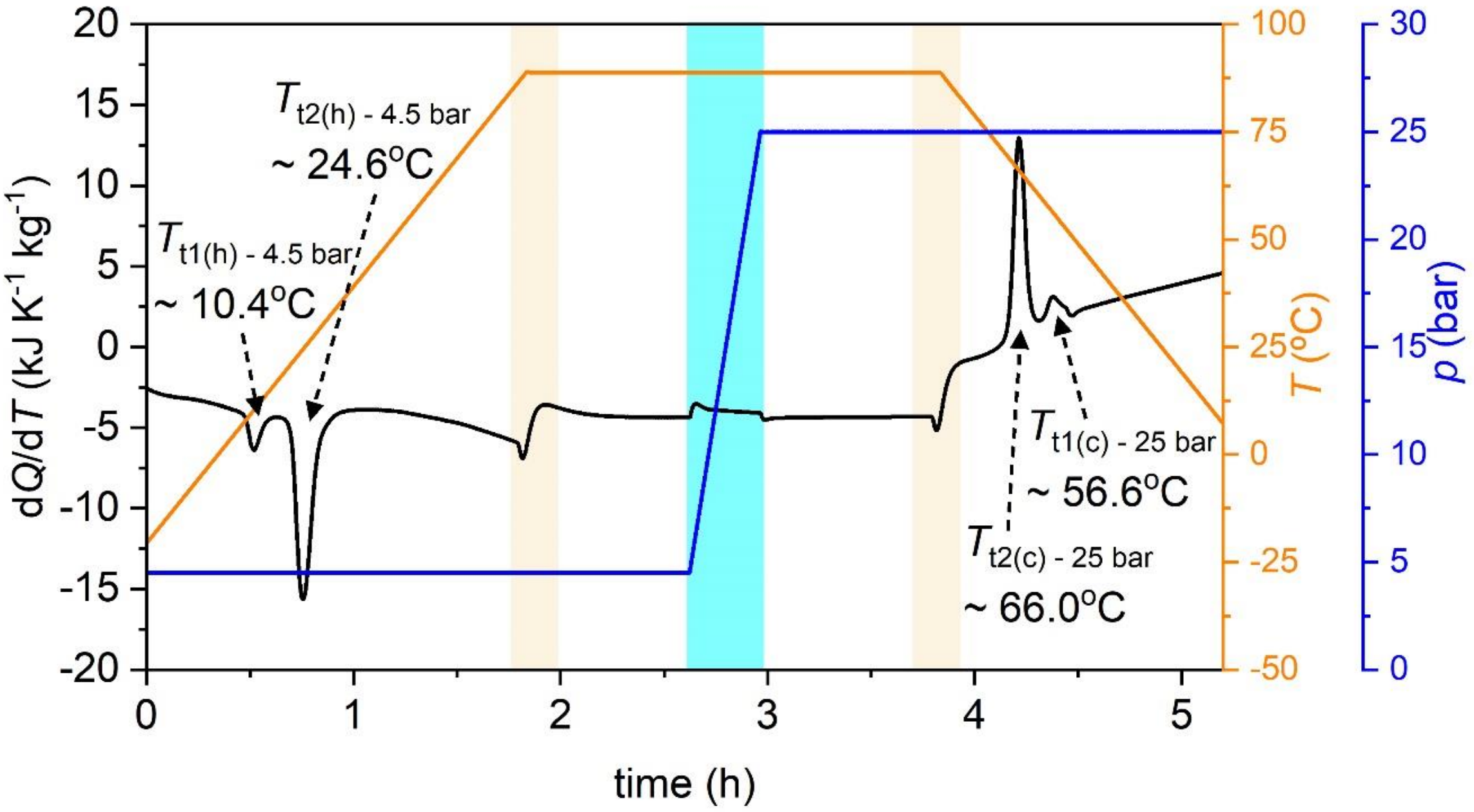

Figure S10. Continuous VT-DSC cycle using different pressures for the heating (energy charge process) and cooling ramp (energy discharge process), $p_{charge}<p_{discharge}$, where the pressure is isothermally increased after the heating ramp. Note: d*Q*/d*T* over time: black solid line, *T* over time: orange solid line, *p* over time: blue solid line. Yellow shaded areas highlight changes in d*Q*/d*T* attributed to changes in the temperature ramp. Blue shaded area indicates changes in d*Q*/d*T* attributed to the CO_2 isothermal pressurization.

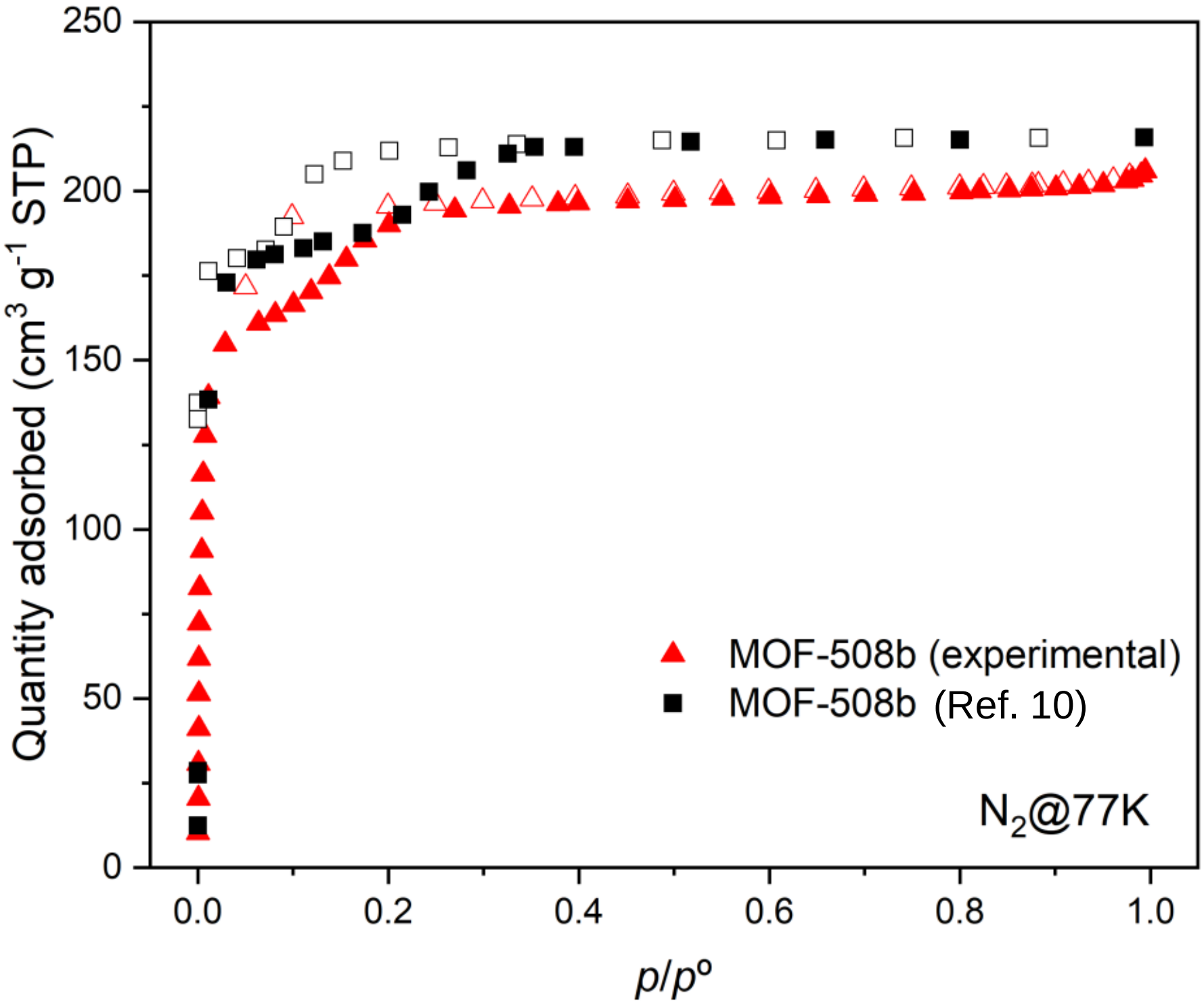

Figure S11. Experimental nitrogen sorption isotherms at 77 K of the MOF-508b in comparisson with values reported in reference [10]. Note: filled symbols indicate adsorption data and open symbols indicate desorption data.

References:

(1) Coudert, F.-X.; Jeffroy, M.; Fuchs, A. H.; Boutin, A.; Mellot-Draznieks, C. Thermodynamics of Guest-Induced Structural Transitions in Hybrid Organic - Inorganic Frameworks. *J. Chem. Soc.* **2008**, *130*, 14294–14302. https://doi.org/10.1021/ja805129c.

(2) Boutin, A.; Springuel-Huet, M.-A.; Nossov, A.; Gédéon, A.; Loiseau, T.; Volkringer, C.; Férey, G.; Coudert, F.-X.; Fuchs, A. H. Breathing Transitions in MIL-53 (Al) Metal–Organic Framework Upon Xenon Adsorption. *Angew. Chemie - Int. Ed.* **2009**, *48*, 8314–8317. https://doi.org/10.1002/anie.200903153.

(3) Boutin, A.; Coudert, F.-X.; Springuel-Huet, M.-A.; Neimark, A. V; Férey, G.; Fuchs, A. H. The Behavior of Flexible MIL-53(Al) upon CH4 and CO2 Adsorption. *J. Phys. Chem. C* **2010**, *114*, 22237–22244. https://doi.org/10.1021/jp108710h.

(4) Bhatt, P. M.; Batisai, E.; Smith, V. J.; Barbour, L. J. Creation of New Guest Accessible Space under Gas Pressure in a Flexible MOF: Multidimensional Insight through Combination of in Situ Techniques. *Chem. Commun.* **2016**, *52* (76), 11374–11377. https://doi.org/10.1039/c6cc06709c.

(5) Bonneau, M.; Lavenn, C.; Zheng, J. J.; Legrand, A.; Ogawa, T.; Sugimoto, K.; Coudert, F. X.; Reau, R.; Sakaki, S.; Otake, K. ichi; Kitagawa, S. Tunable Acetylene Sorption by Flexible Catenated Metal–Organic Frameworks. *Nat. Chem.* **2022**, *14* (7), 816–822. https://doi.org/10.1038/s41557-022-00928-x.

(6) Lloveras, P.; Aznar, A.; Barrio, M.; Negrier, P.; Popescu, C.; Planes, A.; Mañosa, L.; Stern-Taulats, E.; Avramenko, A.; Mathur, N. D.; Moya, X.; Tamarit, J. L. Colossal Barocaloric Effects near Room Temperature in Plastic Crystals of Neopentylglycol. *Nat. Commun.* **2019**, *10* (1), 1–7. https://doi.org/10.1038/s41467-019-09730-9.

(7) Li, J.; Barrio, M.; Dunstan, D. J.; Dixey, R.; Lou, X.; Tamarit, J. L.; Phillips, A. E.; Lloveras, P. Colossal Reversible Barocaloric Effects in Layered Hybrid Perovskite (C10H21NH3)2MnCl4 under Low Pressure Near Room Temperature. *Adv. Funct. Mater.* **2021**, *31* (46), 2105154.

(8) Ferrer, G.; Barreneche, C.; Solé, A.; Martorell, I.; Cabeza, L. F. New Proposed Methodology for Specific Heat Capacity Determination of Materials for Thermal Energy Storage (TES) by DSC. *J. Energy Storage* **2017**, *11*, 1–6. https://doi.org/10.1016/j.est.2017.02.002.

(9) Barthelet, K.; Marrot, J.; Riou, D.; Férey, G. A Breathing Hybrid Organic-Inorganic Solid with Very Large Pores and High Magnetic Characteristics. *Angew. Chemie - Int. Ed.* **2002**, *41* (2), 281–284. https://doi.org/https://doi.org/10.1002/1521-3773(20020118)41:2<281::AID-ANIE281>3.0.CO;2-Y.

(10) Chen, B.; Liang, C.; Yang, J.; Contreras, D. S.; Clancy, Y. L.; Lobkovsky, E. B.; Yaghi, O. M.; Dai, S. A Microporous Metal–Organic Framework for Gas-Chromatographic Separation of Alkanes. *Angew. Chemie - Int. Ed.* **2006**, *45*, 1390. https://doi.org/https://doi.org/10.1002/anie.200502844.
